# Estimation of Grain Yield and Quality in Awned and Awnless Wheat Genotypes Using UAV and Proximal Multispectral Sensors

**DOI:** 10.3390/plants15182819

**Published:** 2026-09-14

**Authors:** Irina Marina Stević, Vesna Kandić Raftery, Marko Kostić, Nataša Ljubičić, Biljana Bošković, Kosta Gligorević, Miloš Pajić, Milan Dražić

**Affiliations:** 1 Institute of Agricultural Economics, Volgina 15, 11060 Belgrade, Serbia; 2 Maize Research Institute, Zemun Polje, Slobodana Bajića 1, 11185 Belgrade, Serbia; 3 Faculty of Agriculture, University of Novi Sad, Trg Dositeja Obradovića 8, 21000 Novi Sad, Serbia; 4 Biosense Institute, University of Novi Sad, Dr Zorana Đinđića 1, 21000 Novi Sad, Serbia; 5 Faculty of Agriculture, University of Belgrade, Nemanjina 6, 11080 Belgrade, Serbia

**Keywords:** vegetation indices, remote sensing, precision agriculture, NDVI, GNDVI, NDRE

## Abstract

This study aimed to evaluate the complementary potential of UAV-based and proximal multispectral sensing using the Plant-O-Meter (POM) sensor for the assessment of wheat grain yield and quality across different phenological stages and two growing seasons. The analysis was based on three vegetation indices, the Normalized Difference Vegetation Index (NDVI), Green Normalized Difference Vegetation Index (GNDVI), and Normalized Difference Red Edge Index (NDRE), and included two morphologically distinct genotype groups, awned and awnless. The study included nine awned and nine awnless genotypes. Vegetation indices showed pronounced seasonal dynamics, with higher values during intensive vegetative development and a decline during later growth stages. The strongest relationship with grain yield was observed for GNDVI-A during the heading to beginning of flowering stage (BBCH 51–61) in Season I (r = 0.85), whereas in Season II, the strongest relationship was observed for NDRE-A during the flag leaf stage (BBCH 37–39) (r = 0.80). Awned genotypes generally showed stronger VI–yield relationships, while associations with grain quality parameters varied among genotype groups and seasons. Linear mixed-effects models showed that both VI-related effects and genotype variability contributed to the variation in grain yield and quality. For yield, marginal R^2^ was 0.43 in Season I and 0.50 in Season II, while conditional R^2^ was 0.78 and 0.64, respectively. For protein and wet gluten content, model performance was more variable, with marginal R^2^ values ranging from 0.30 to 0.39 for protein and from 0.31 to 0.33 for wet gluten. Genotype-level LOGO cross-validation further indicated variation in model performance when genotypes not included in model development were evaluated. Overall, the results indicate that relationships between multispectral vegetation indices and wheat grain yield and quality depend on sensing method, phenological stage, genotype characteristics, and growing season. The findings provide an exploratory basis for the application of multispectral sensing in wheat phenotyping and assessment of grain yield and quality, while further validation across broader genetic and environmental conditions is required.

## 1. Introduction

Wheat (*Triticum aestivum* L.) is one of the most important crops in the world and plays a crucial role in global food production. Its production has strategic importance both at the national level and for the global economy. In addition to grain yield, wheat quality parameters, particularly protein content and wet gluten content, have a major influence on the technological and nutritional value of wheat grain [1].

However, being cultivated under diverse climatic conditions, wheat productivity depends on several factors, including genetic potential, agronomic management practices, and especially soil properties and climate conditions. In recent years, global wheat production has been increasingly affected by climate change, including more frequent and severe drought stress events, which is why precise and periodic monitoring of crop characteristics becomes necessary [2,3,4,5]. Research in the field of precision agriculture, supported by advanced remote sensing technologies, enables precise monitoring and assessment of vegetation status, which is essential for optimizing agricultural production and ensuring stable crop yields [6].

In this research, the analyzed wheat genotypes were divided into two groups based on the presence or absence of awns, as these two morphological types of wheat differ in their physiological responses to abiotic stress. Awns contribute to spike photosynthesis and may play a significant role under drought and high-temperature conditions, since the photosynthetic activity of spike tissues in awned genotypes decreases to a lesser extent than that of leaves, while the contribution of spike photosynthesis to yield formation increases significantly, reaching up to 22–45% in conditions of limited assimilate supply [7]. Although the positive effect of awns on grain yield has not been consistently confirmed across different agroecological conditions, there are claims that they can contribute to photosynthetic activity [8,9]. These insights indicate that awned and awnless genotypes represent functionally distinct groups with potentially different canopy responses that can be detected using VIs. This grouping was considered appropriate to reduce variability associated with physiological differences and to enable more precise comparisons across different stages of plant development.

The application of precision agriculture encompasses various advanced technologies that enable more efficient, sustainable and responsible management of agricultural resources [10]. Remote sensing represents an integral component of precision agriculture. The development of remote and proximal sensing technologies has made it possible to effectively monitor the condition of crops during the growing season [11], providing detailed insights into the physiological condition of plants even before visible symptoms of stress occur. Consequently, remote sensing methods have become highly valuable tools for early detection of plant stress, optimization of crop nutrition, and yield assessment [12,13].

Recent research highlights the growing importance of integrating complementary biological and technological approaches for crop monitoring and protection, including studies of molecular mechanisms underlying plant disease resistance [14] and advanced computer vision methods for fine-grained agricultural pest detection and segmentation [15]. Within this broader development of precision agriculture, vegetation indices (VIs) have become important tools for the quantitative assessment of crop condition [16].

In wheat, indices such as NDVI (Normalized Difference Vegetation Index), GNDVI (Green Normalized Difference Vegetation Index), and NDRE (Normalized Difference Red Edge Index) provide important information regarding the overall condition of the crop. These VIs enable continuous monitoring of wheat throughout the entire growing season and across different growth and development stages, and they are widely used as indicators of photosynthetic activity, chlorophyll content, and general plant health status. VIs allow indirect estimation of grain yield and grain quality, which is of particular importance for timely decision-making in wheat production [17,18].

Among VIs, NDVI is the most commonly used index and, during specific stages of plant development, it has shown statistically highly significant correlations with grain yield [19,20]. NDVI effectively reflects photosynthetic activity, enabling the detection of most deficiencies associated with physiological performance [16]. NDVI values are widely used for continuous crop monitoring and the assessment of grain yield under different agroclimatic conditions [21,22,23,24,25].

Previous studies [26,27] have shown that GNDVI exhibits complex relationships with climatic changes and yield. In several studies, GNDVI proved to be more effective than NDVI, demonstrating a higher coefficient of determination with yield and lower model error [13,28,29]. This may be explained by the greater sensitivity of GNDVI to subtle changes in nitrogen content and chlorophyll concentration in plants. For this reason, GNDVI is frequently used to monitor the nutritional status of crops.

An analysis of the chlorophyll content in plants showed that NDRE is most effective; it is most useful from the middle of the growing season, during stem elongation (BBCH 31–32) and the end of stem elongation (BBCH 37–39), to harvest [30]. VIs such as NDVI and GNDVI are effective for evaluating and monitoring photosynthetic activity, whereas newer indices such as NDRE have the ability to detect subtle crop variations during specific growth stages [30,31].

New developments have pushed the capabilities of Unmanned Aerial Vehicle (UAV) platforms from traditional data gathering to the area of more reliable localization and autonomous operation. For instance, CPFL has developed a vision-based method for achieving continuous UAV localization in situations where GNSS signals are not available, while HTGL has concentrated on robust visual geo-localization during oblique flight and in complex scenes [32,33]. These advances show the wider trend of UAV platforms moving towards more reliable and autonomous performance. In the field of agriculture, UAVs allow for the quick and repeated collection of high-resolution spatial data, giving detailed information on crop conditions over the entire growing season [34,35,36,37]. Proximal sensors, on the other hand, make it possible to obtain more detailed measurements at the crop canopy level. The combination of these complementary sensing technologies enables a more thorough assessment of crop variability, improves the accuracy of models used to estimate agronomic parameters, and supports crop optimization [38,39,40]. Combining UAV and proximal sensing with accurate yield and grain quality measurements can also help in understanding the complex relationships between crop performance and production characteristics.

Previous research has demonstrated that statistical analyses, particularly correlation analysis, can reveal strong connections between specific VIs and crop yield [41,42,43,44]. In addition, linear regression analysis is widely applied in order to quantify the assessment potential of VIs in the estimation of agronomic parameters. Descriptive statistical methods, as well as correlation analyses between VIs and grain yield, are frequently used to investigate the relationship between spectral data, crop productivity, and wheat grain quality. These approaches provide a more detailed understanding of how variations in plant reflectance are associated with chlorophyll content, crop development, and the potential to achieve higher grain yields and improved grain quality.

Although VIs derived from UAVs or proximal sensors have been widely investigated for wheat monitoring, studies directly comparing the UAV-based DJI Phantom 4 Multispectral and the proximal Plant-O-Meter (POM) sensor under identical field conditions remain very limited. The Plant-O-Meter sensor represents a relatively recent proximal multispectral sensing technology whose application in wheat phenotyping and grain quality estimation has received only limited attention in the international literature. Furthermore, most previous studies have evaluated sensing platforms independently and across a limited number of phenological stages, without systematically considering the interaction between sensor type, vegetation index, phenological stage, genotype morphology, and environmental conditions. The present study addresses this gap by simultaneously evaluating these factors within a single integrated experimental framework across two contrasting growing seasons and two morphologically distinct wheat genotype groups (awned and awnless). Rather than identifying a single universally superior sensing strategy, the results demonstrate that estimation performance is strongly dependent on the interaction among sensor type, vegetation index, phenological stage, and environmental conditions, a finding with direct practical relevance for the design of remote sensing-based crop monitoring and phenotyping programs.

The aim of this research was to evaluate the applicability and efficiency of VIs (NDVI, GNDVI and NDRE) obtained through UAV and POM measurements for the estimation of wheat grain yield and grain quality parameters (protein and wet gluten content), as well as to determine differences in their efficiency between awned and awnless wheat genotypes during different developmental stages. Additionally, the stability and reliability of the obtained regression models were assessed through a validation procedure, which confirmed their ability to estimate grain yield and grain quality parameters from left-out observations.

## 2. Results

The results of the statistical analyses conducted in accordance with the described methodology are presented in this section. The analysis includes the variability in the observed parameters (grain yield, protein content (PC) and wet gluten content (WGC)), VI values, and mutual relationships between the examined traits. Particular emphasis was placed on the identification of significant correlations between VI values and grain yield, protein content, and wet gluten content, as well as on the evaluation of their significance through linear regression analysis. The results are presented by season, development stage (based on the BBCH scale), and analyzed genotype group in order to provide a clearer understanding of the observed differences and patterns of crop response. VIs marked with the letter “A” represent VIs obtained for awned genotypes.

In order to evaluate the variability in the investigated parameters, Table 1 presents the basic descriptive statistics for grain yield, protein content, and wet gluten content in awned and awnless genotypes during the I and II seasons. The presented parameters include mean values (Mean), standard deviation (SD), and coefficient of variation (Cv %) for each investigated trait.

The differences in wheat yield between growing seasons indicate a significant influence of climatic conditions (precipitation and temperature). During the I season, a precipitation deficit occurred in critical phenological stages of crop development, which limited yield potential despite the application of identical agronomic practices. In contrast, the more favorable water regime during the II season contributed to more intensive crop growth and improved realization of genetic yield potential. Differences were observed between awned and awnless genotypes. Awned genotypes achieved slightly higher average grain yield values in both seasons, whereas during the I season, awnless genotypes exhibited higher values of protein and wet gluten content.

### 2.1. Descriptive Statistics for Season I

The descriptive statistics of VI values obtained through UAV measurement during the I season revealed marked seasonal dynamics and relatively stable VI values (Table 2). The mean values of the evaluated VIs reached their maximum during the flag leaf to end of stem elongation stage (BBCH 37–39), after which a sharp decline was observed. The standard deviation values of the VIs were relatively low during the middle of the vegetation stage (stem elongation BBCH 31–32, SD ≈ 0.03), which reflects the uniform development of the crop, while higher values of the VIs were recorded in the initial (tillering BBCH 21–25) and late growth stages (wax maturity BBCH 85–87, SD ≈ 0.05).

The coefficient of variation further confirmed the observed pattern of standard deviation, with the lowest values recorded during the stage of maximum crop development (flag leaf to the end of stem elongation, BBCH 37–39) when NDVI Cv ≈ 3.5%. Contrary to these findings, substantially higher values were observed during the later developmental stages, particularly during wax maturity (BBCH 85–87), when the coefficient of variation increased up to 23.25%.

Building on the results obtained from UAV measurements, descriptive statistical analysis of VIs measured using the POM device during the I season confirmed similar seasonal dynamics, although with more noticeable fluctuations in certain developmental stages. The highest mean values of the VIs (Table 3) were recorded during stem elongation (BBCH 31–32).

The standard deviation indicated a relatively homogeneous wheat crop during active growth stages (from the beginning of stem elongation to the end of stem elongation, BBCH 29–39) (SD ≈ 0.03–0.05), while higher standard deviation values were observed in the later stages (wax ripeness, BBCH 85–87), indicating pronounced wheat heterogeneity during maturation. The coefficient of variation further confirmed this pattern, with low values during the stem elongation stage (BBCH 31–32) ≈ 4–5%, whereas in the wax ripeness stage (BBCH 85–87), the coefficient of variation reached very high values (up to ≈39%).

Overall, the results obtained using UAV and POM measurements showed a high level of agreement regarding the seasonal character of VI values, whereby UAV measurements provided more stable and more uniform VI values, while POM measurements registered more pronounced local differences. This can be explained by the data acquisition approach, where UAV-derived VI values represent a spatial average of a large number of pixels, in contrast to POM measurements, which include a limited number of measurements per observed plot, potentially leading to greater data variability.

### 2.2. Descriptive Statistics for Season II

In the II season, a similar trend was observed for the analyzed VIs, with some changes in variability. Descriptive statistics of VI values obtained from UAV measurements indicated pronounced stability and high VI values throughout most of the growing season (Table 4). Mean VI values reached high levels at the tillering stage (BBCH 21–25), with maximum values recorded during stem elongation (BBCH 31–32), followed by a slight decline toward the heading–early flowering stage (BBCH 51–61) and a sharp decrease in the final stage of vegetation (BBCH 71–75).

The standard deviation values of VIs were low during most of the growing season (SD ≈ 0.01–0.03), indicating high crop uniformity. Also, the coefficient of variation increased only in the later growth stages (BBCH 85–87), reaching a value of up to approximately 11%. Compared to the I season, greater homogeneity in the observed genotypes was detected during the II season. UAV-derived VI data for the II season indicated a more stable wheat genotype development and lower spatial variability.

Descriptive analysis of VI values in the II season obtained using POM measurements indicated a similar trend to that observed for VI values derived from UAV measurements. Mean VI values reached their maximum during the transition from tillering to stem elongation (BBCH 29–30), while only the mean NDRE value of awned genotypes reached its maximum during the stem elongation stage (BBCH 31–32). In the final growth stages (grain filling, BBCH 71–75), a sharp decline in NDVI values was recorded, while GNDVI and NDRE values also decreased (Table 5).

The coefficient of variation remained relatively stable throughout most phenological stages (Cv 4–8%), except during the wax ripeness stage (BBCH 71–75), where a significant increase was noted. The maximum values of most VIs during the II season were recorded at an earlier phenological stage. More specifically, VIs obtained from UAV measurements in the II season reached their maximum during stem elongation (BBCH 31–32; NDVI–0.90), whereas in the I season, their maximum values were reached in the subsequent phenological stage, the leaf emergence end of stem elongation (BBCH 37–39; NDVI–0.84).

The maximum VI values obtained from POM measurements showed the same trend. In the II season, the maximum VI values were reached during the end of tillering–beginning of stem elongation stage (BBCH 29–30; NDVI–0.77), while in the I season, the maximum values were recorded during stem elongation (BBCH 31–32; NDVI–0.76).

### 2.3. Correlation Coefficients Between VIs (UAV and POM, Season I) and Yield, PC and WGC

**Figure 1:**
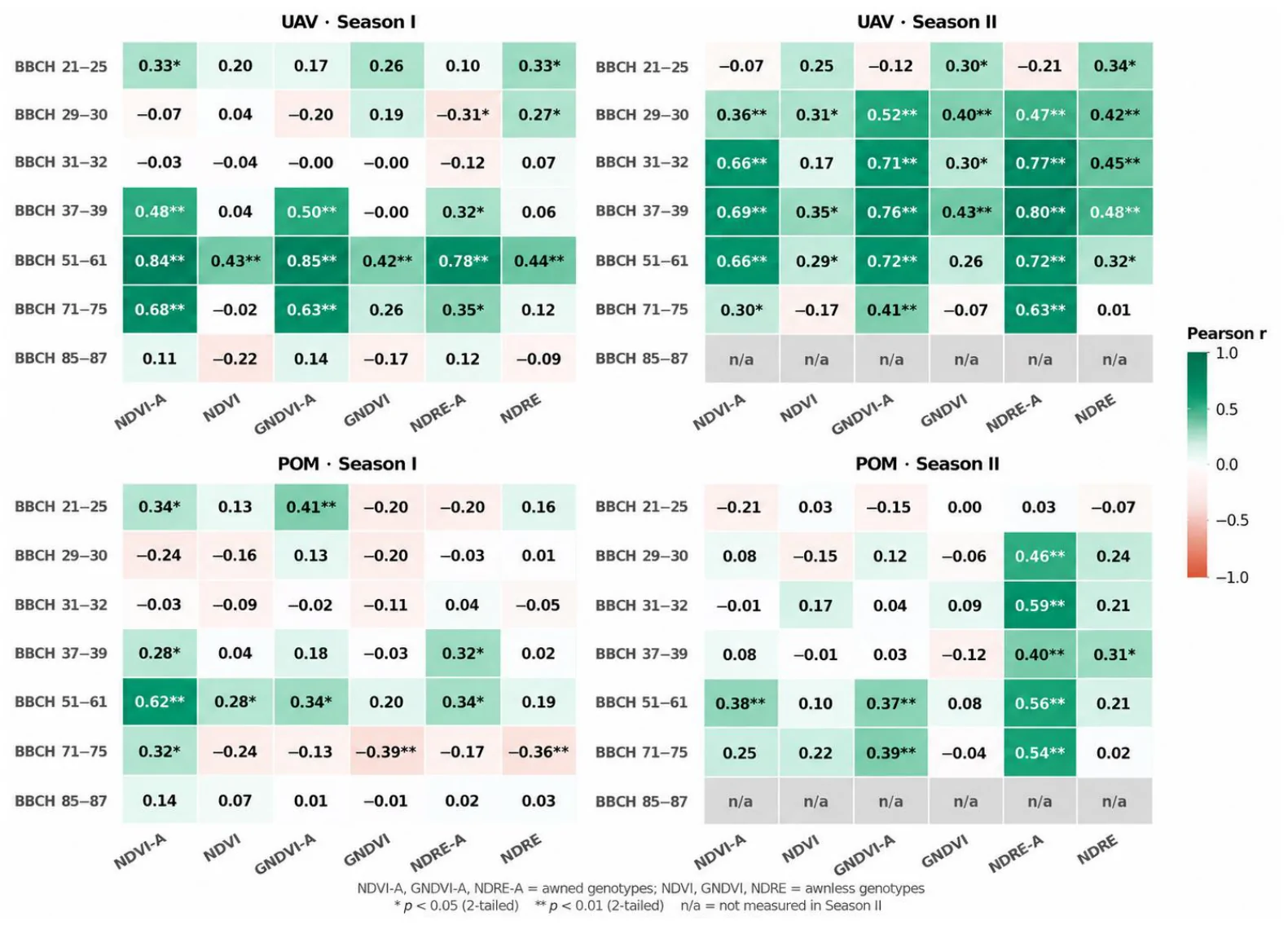
Correlation coefficients between yield and VIs during Seasons I and II.

**Figure 2:**
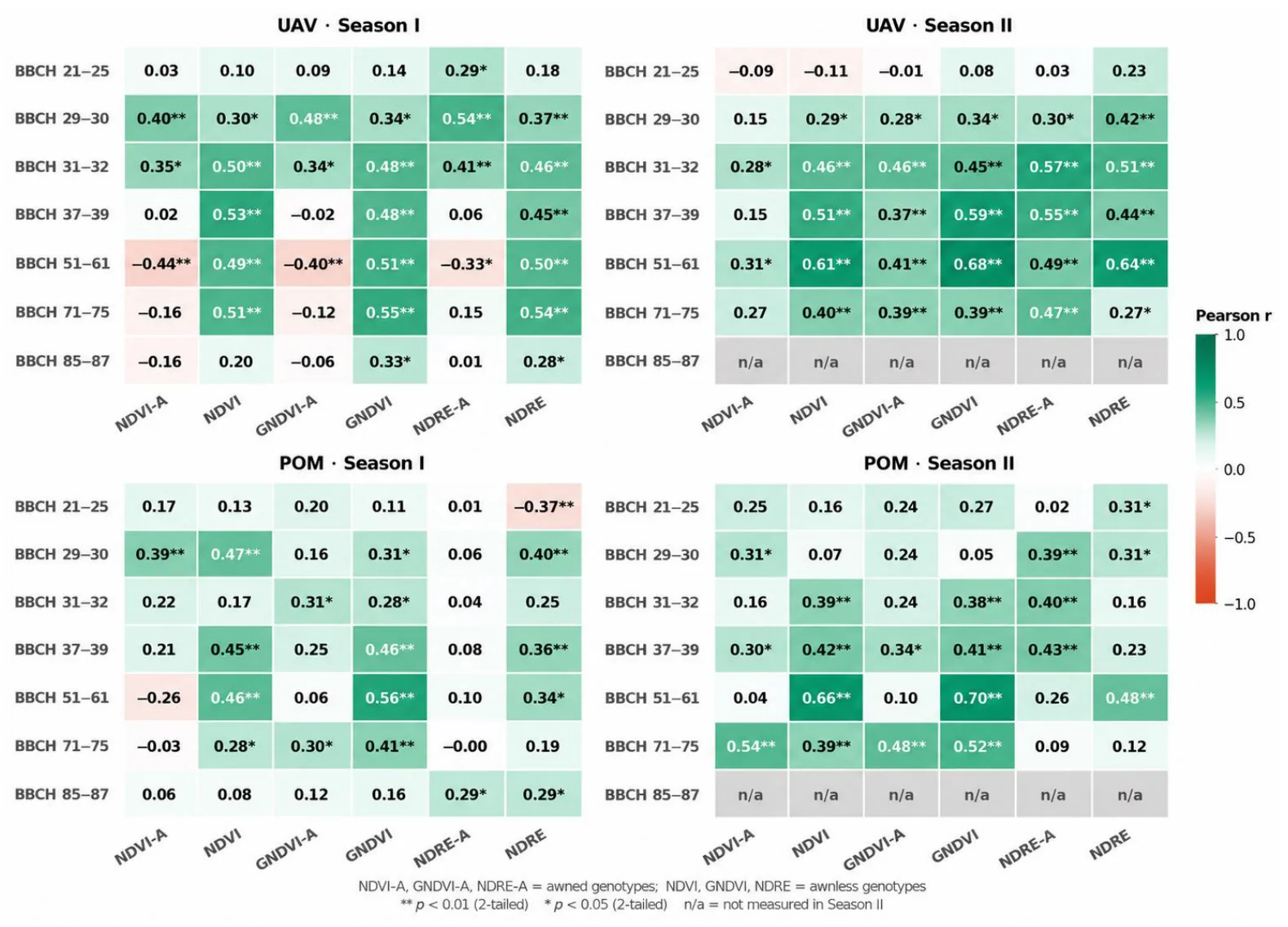
Correlation coefficients between protein content and VIs during Seasons I and II.

**Figure 3:**
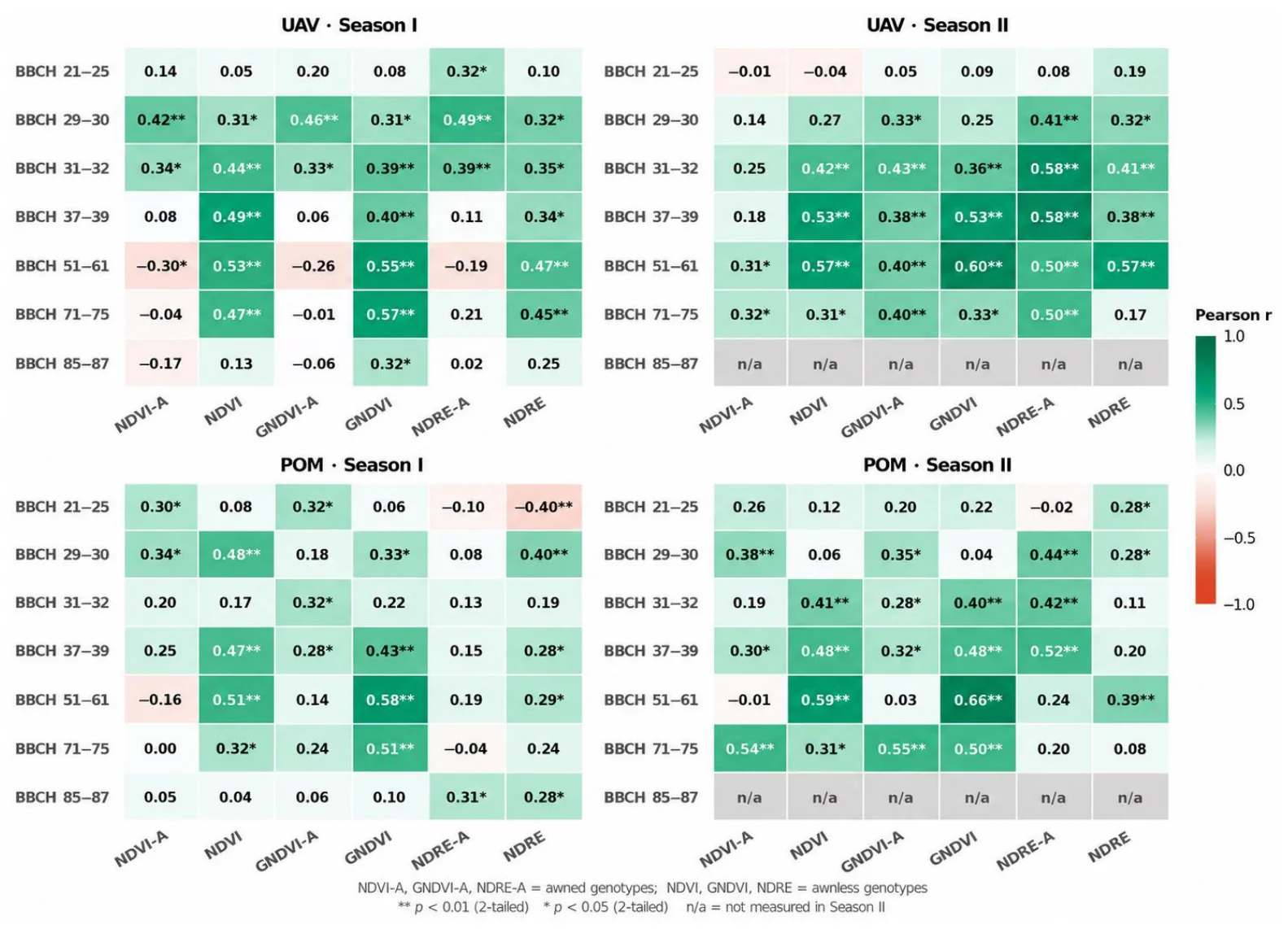
Correlation coefficients between wet gluten content and VIs during Seasons I and II.

Strong and statistically significant relationships were examined between VI values obtained from the UAV measurements and analyzed variables (yield, PC and WGC; Figure 1, Figure 2 and Figure 3), with the highest correlation values recorded during the heading–beginning of flowering stage (BBCH 51–61) and grain filling stage (BBCH 71–75). The most significant positive correlations with yield (Figure 1) were achieved by VI values obtained from UAV measurements of awned genotypes, where very high correlation values were recorded during the heading–beginning of flowering stage (BBCH 51–61), namely, GNDVI—A (r = 0.85), NDVI—A (r = 0.84) and NDRE—A (r = 0.78).

On the other hand, wheat grain quality parameters showed the strongest and most consistent correlations with VI values (UAV measurements) in awnless genotypes (Figure 2 and Figure 3), particularly during the grain filling stage (BBCH 71–75), where high correlation values were recorded between the GNDVI value and protein content (r = 0.55, Figure 2), as well as between the NDRE value and protein content (r = 0.54).

The strongest correlation between wet gluten content and GNDVI (UAV measurement) was recorded during the grain filling stage (BBCH 71–75; r = 0.57, Figure 3). Significant positive correlations were also observed during the intermediate growth stage (up to r 0.50), confirming that VI values reliably reflect the relationship between the physiological status of plants and the wet gluten content in wheat grains.

Consistent with the previously analyzed data, correlations between VIs derived from UAV and POM measurements showed similar patterns. In awned genotypes, positive correlations with yield were observed in certain growth stages, such as heading–beginning of flowering (BBCH 51–61), where NDVI—A showed a correlation coefficient of r = 0.62 (Figure 1).

In awnless genotypes, the correlation between VI values and quality parameters (protein and wet gluten content) remained evident, particularly during the heading–beginning of flowering stage (BBCH 51–61), with correlations observed between GNDVI and protein content (r = 0.56, Figure 2), as well as between GNDVI and wet gluten content (r = 0.58, Figure 3).

### 2.4. Correlation Coefficients Between VIs (UAV and POM, Season II) and Yield, PC and WGC

Correlation analysis between VI values obtained from UAV measurements and quality parameters (protein and wet gluten content) and yield in the II season indicates clear differences between awned and awnless genotypes. As in the I season, the VI values obtained from UAV measurements of awned genotypes showed more pronounced and stronger correlations with yield (Figure 1). Particularly high correlations were observed during the intermediate growth stage, such as the correlation between NDRE—A and yield (r = 0.80) and GNDVI—A and yield (r = 0.76) during the flag–end of stem elongation stage (BBCH 37–39). Significant correlation values were also recorded during the stem elongation stage (BBCH 31–32), where the correlation between NDRE—A and yield reached r = 0.77, confirming that VI values in awned genotypes better reflect yield variability.

Awnless genotypes showed more stable and stronger positive correlations with quality parameters (protein and wet gluten content), particularly during the stage of intensive growth (from the beginning of stem elongation to heading BBCH 29–61), where high and statistically significant values were recorded. These included correlations between NDVI and protein content (r = 0.61) and between GNDVI and protein content (r = 0.68) during the heading–beginning of flowering stage (BBCH 51–61, Figure 2), as well as correlations between NDRE and wet gluten content (r = 0.57) and between GNDVI and wet gluten content (0.60, Figure 3). These results indicate a stronger relationship between VI values and grain quality parameters (protein and wet gluten content) in awnless genotypes. Within this method, NDRE stood out as the most reliable VI, as it achieved the highest correlation values with the analyzed parameters, particularly with yield in awned genotypes and with quality parameters (protein and wet gluten content) in awnless genotypes. In the later vegetation stages (grain filling and wax ripeness, BBCH 71–87), these correlations weakened.

Analogous to the remote sensing results obtained using UAV measurements, VI values obtained from POM measurements in the II season also confirmed clear relationships between VIs and quality parameters (protein and wet gluten content) and yield. In certain phenological stages, these relationships were more pronounced and specific. In awned genotypes, stronger correlations between VIs and yield were observed (Figure 1), with NDRE—A particularly standing out due to statistically significant correlations across several stages of development, including stem elongation (BBCH 31–32), with r = 0.59, heading–beginning of flowering (BBCH 51–61), with r = 0.56, and earlier stages (beginning of stem elongation, BBCH (29–30)), with r = 0.46.

VIs measured using the POM device in awnless genotypes showed the most noticeable correlations with quality parameters (protein and wet gluten content), particularly during the heading–beginning of flowering stage (BBCH 51–61), where high correlation values were recorded between GNDVI and protein content (r = 0.70) and NDVI and protein content (r = 0.66, Figure 2), as well as between GNDVI and wet gluten content (r = 0.66) and NDVI and wet gluten content (r = 0.59, Figure 3).

### 2.5. Linear Mixed-Effects Regression Analysis

Based on the previously conducted correlation analysis, which included VI values obtained using both the POM and UAV methods, the vegetation indices showing the strongest statistically significant relationships with the analyzed wheat traits (grain yield, protein content, and wet gluten content) were identified and selected for further analysis. To further evaluate the relationships between the selected vegetation indices and the analyzed wheat traits while accounting for the hierarchical structure of the data, linear mixed-effects models were applied. The vegetation index was included as a fixed effect, whereas genotype was included as a random effect to account for the non-independence of observations arising from repeated measurements of the same genotypes. The models were used to estimate the effect of each selected vegetation index on the respective wheat trait and to assess its statistical significance and model performance.

#### 2.5.1. Estimation of Wheat Yield Using VIs

For yield estimation during the I season, GNDVI-A values obtained from the UAV measurements of awned genotypes during the heading to beginning of flowering stage (BBCH 51–61) were selected, as this stage showed the strongest positive correlation with yield (r = 0.85; Figure 1). The linear mixed-effects model indicated a statistically significant relationship between GNDVI-A and yield (F = 16.81; *p* = 7.20 × 10^−4^). The marginal coefficient of determination (R^2^m = 0.43) indicated that the fixed effect of GNDVI-A explained 43% of the variability in yield, while the conditional R^2^ (R^2^c = 0.78) showed that the regression model, including the variability accounted for by the random effect of genotype, explained 78% of the total variability. The estimated GNDVI-A coefficient was β = 10,453.92 kg∙ha^−1^, with a 95% confidence interval ranging from 5456.00 to 15,451.60 kg∙ha^−1^ (Table 6).

The estimated fixed-effect coefficient indicated that an increase in GNDVI-A of 0.1 units was associated with an average increase in yield of approximately 1045.39 kg∙ha^−1^. The model showed good estimation performance, with an RMSE of 349.79 kg∙ha^−1^, an MAE of 276.68 kg∙ha^−1^, a relative error of 5.72%, a bias of 0, an NSE of 0.88, and Lin’s CCC of 0.93 (Table 7).

For yield estimation during the II season, NDRE-A values of awned genotypes were selected, as UAV measurements showed the strongest positive correlation with yield during the flag leaf emergence to the end of stem elongation stage (BBCH 37–39; r = 0.80; Figure 1). The linear mixed-effects model indicated a statistically significant relationship between NDRE-A and yield (F = 28.44; *p* = 4.53 × 10^−5^; Table 6). The marginal coefficient of determination (R^2^m = 0.50) indicated that the fixed effect of NDRE-A explained 50% of the yield variability, while the conditional R^2^ (R^2^c = 0.64) showed that the model, including the random effect of genotype, explained 64% of the total variability.

The estimated fixed-effect coefficient was β = 30,464.56 kg∙ha^−1^, with a 95% confidence interval ranging from 19,269.20 to 41,659.92 kg∙ha^−1^. This indicates that an increase in NDRE-A of 0.01 units was associated with an average increase in yield of approximately 304.65 kg∙ha^−1^. The model showed good estimation performance, with RMSE = 674.08 kg∙ha^−1^, MAE = 493.12 kg∙ha^−1^, relative error = 4.54%, bias = 0, NSE = 0.76, and Lin’s CCC = 0.86 (Table 7).

The corresponding regression plots with 95% prediction intervals for both seasons are provided in the Supplementary Materials (Figure S1).

#### 2.5.2. Estimation of Wheat Protein Content Using VIs

During the I season, a strong positive correlation was found between protein content and GNDVI values in awnless genotypes based on POM measurements during the heading to beginning of flowering stage (BBCH 51–61; r = 0.56; Figure 2). Based on this relationship, GNDVI was selected for further estimation of protein content using a linear mixed-effects model. The regression model showed a statistically significant relationship between GNDVI and protein content (F = 15.06; *p* = 4.56 × 10^−4^; Table 6). The marginal coefficient of determination (R^2^m = 0.30) indicated that the fixed effect of GNDVI explained 30% of the variability in protein content, while the conditional R^2^ (R^2^c = 0) indicated that the inclusion of the random effect of genotype did not provide an additional contribution to explaining model variability.

The estimated fixed-effect coefficient was β = 8.96%, with a 95% confidence interval ranging from 4.43 to 13.48%. This indicates that an increase in GNDVI of 0.1 units was associated with an average increase in protein content of approximately 0.90%. The model showed an RMSE of 0.65%, an MAE of 0.53%, a relative error of 4.94%, bias = 0, NSE = 0.31, and Lin’s CCC = 0.47 (Table 7).

During the II season, a strong positive correlation was found between protein content and GNDVI values in awnless genotypes, based on POM measurements during the heading to beginning of flowering stage (BBCH 51–61; r = 0.56; Figure 2). Based on this relationship, GNDVI was selected for further estimation of protein content using a linear mixed-effects model. The analysis showed a statistically significant relationship between GNDVI and protein content (F = 31.54; *p* = 3.35 × 10^−6^; Table 6). The marginal coefficient of determination (R^2^m = 0.39) indicated that the fixed effect of GNDVI explained 39% of the variability in protein content, while the conditional R^2^ (R^2^c = 0.74) showed that the model, including the random effect of genotype, explained 74% of the total variability. The estimated fixed-effect coefficient was β = 19.99%, with a 95% confidence interval ranging from 13.02 to 26.98%. This indicates that an increase in GNDVI of 0.1 units was associated with an average increase in protein content of approximately 2.00%. The model showed good estimation performance, with RMSE = 0.46%, MAE = 0.39%, relative error = 3.43%, bias = 0, NSE = 0.81, and Lin’s CCC = 0.89 (Table 7).

The corresponding regression plots with 95% prediction intervals for both seasons are provided in the Supplementary Materials (Figure S2).

#### 2.5.3. Estimation of Wheat Wet Gluten Content Using VIs

For the next model, the relationship between GNDVI values obtained from POM measurements during the heading to beginning of flowering stage (BBCH 51–61) and wet gluten content in awnless wheat genotypes during the I season was analyzed (r = 0.56; Figure 3). The linear mixed-effects analysis showed a statistically significant relationship between GNDVI and wet gluten content (F = 15.65; *p* = 4.56 × 10^−4^; Table 6). The marginal coefficient of determination (R^2^m = 0.33) indicated that the fixed effect of GNDVI explained 33% of the variability in wet gluten content, while the conditional R^2^ (R^2^c = 0.41) showed that the model, including the random effect of genotype, explained 41% of the total variability.

The estimated fixed-effect coefficient was β = 31.30%, with a 95% confidence interval ranging from 15.79 to 46.80%. This indicates that an increase in GNDVI of 0.1 units was associated with an average increase in wet gluten content of approximately 3.13 percentage points. The model showed an RMSE of 1.92%, an MAE of 1.52%, a relative error of 7.85%, bias = 0, NSE = 0.45, and Lin’s CCC = 0.60 (Table 7).

During the II season, the relationship between GNDVI values obtained from POM measurements during the heading to beginning of flowering stage (BBCH 51–61) in awnless genotypes and wet gluten content was analyzed (r = 0.56; Figure 3). Considering the close relationship between wet gluten content and the protein complex in wheat grain, it is expected that vegetation indices based on chlorophyll reflectance can also provide an indication of this quality parameter. The linear mixed-effects analysis showed a statistically significant relationship between GNDVI and wet gluten content (F = 22.21; *p* = 4.58 × 10^−5^; Table 6). The marginal coefficient of determination (R^2^m = 0.31) indicated that the fixed effect of GNDVI explained approximately 31% of the variability in wet gluten content, while the conditional R^2^ (R^2^c = 0.70) showed that the model, including the random effect of genotype, explained 70% of the total variability (Table 6).

The estimated fixed-effect coefficient was β = 57.92%, with a 95% confidence interval ranging from 33.83 to 82.09%. This indicates that an increase in GNDVI of 0.1 units was associated with an average increase in wet gluten content of approximately 5.79%. The model showed good estimation performance, with RMSE = 1.59%, MAE = 1.36%, relative error = 5.83%, bias = 0, NSE = 0.78, and Lin’s CCC = 0.87 (Table 7).

The corresponding regression plots with 95% prediction intervals for both seasons are provided in the Supplementary Materials (Figure S3).

The observed-versus-estimated values and residual distributions for the six fitted regression models are presented in the Supplementary Materials (Figure S4), providing a graphical assessment of model fit and residual behavior.

### 2.6. Cross-Validation of Linear Regression Models

To evaluate the robustness and estimation capability of the obtained linear mixed-effects models, a leave-one-genotype-out (LOGO) cross-validation procedure was employed. In each validation iteration, all observations belonging to one genotype were excluded from model fitting, while the model was fitted using the remaining genotypes. The fitted model was then used to estimate the values for the excluded genotype. This procedure was repeated sequentially for each genotype, ensuring that the performance of the models was evaluated on genotypes that were not included in model development. The performance of the models was assessed using the cross-validated coefficient of determination (R^2^LOGO), root mean square error of cross-validation (RMSELOGO), and mean absolute error of cross-validation (MAELOGO). These metrics were used to evaluate the robustness and estimation accuracy of the models when applied to genotypes not included in model fitting.

For yield estimation, the GNDVI-A model in the I season achieved R^2^LOGO = 0.53, with an RMSELOGO of 732.09 kg∙ha^−1^ and an MAELOGO of 597.28 kg∙ha^−1^, whereas the NDRE-A model in the II season achieved R^2^LOGO = 0.51, with an RMSELOGO of 971.58 kg∙ha^−1^ and an MAELOGO of 720.57 kg∙ha^−1^. The results of the leave-one-genotype-out validation for all analyzed models are presented in Table 8.

For protein content estimation, the GNDVI models achieved R^2^LOGO values of 0.26 in the I season and 0.38 in the II season. The RMSELOGO values were 0.67% and 0.84%, while the MAELOGO values were 0.58% and 0.69%, respectively. For wet gluten content, R^2^LOGO was 0.25 in the I season and 0.31 in the II season, with RMSELOGO values of 2.27% and 2.86% and MAELOGO values of 2.00% and 2.45%, respectively.

Overall, the validation results confirm that the models based on the selected vegetation indices retained a certain ability to estimate yield and quality traits when tested on genotypes that were not included in model development. However, the lower R^2^LOGO values compared with the R^2^ values obtained from the models indicate a reduction in performance under the more stringent validation procedure, confirming the importance of genotypic variability and indicating that the results should be interpreted as an assessment of the models’ potential rather than as evidence of their universal applicability.

## 3. Discussion

The results of this research indicate pronounced seasonal dynamics of VI values, with maximum values during the stages of intensive vegetative growth (stem elongation–flag leaf stage, BBCH 31–39) and a decline in the later growth stage (grain filling and wax ripeness, BBCH 71–87), which is consistent with previous research in the field of remote sensing [38]. Such variation in VI values reflects changes in chlorophyll content and plant photosynthetic activity, with VIs reaching their maximum values during stages characterized by maximum leaf area and intensive photosynthetic activity [45]. A particularly important finding relates to the differences observed between the I and II seasons, which may be explained by differences in temperature regimes and precipitation distribution. The lack of precipitation during critical phenological stages in the I season (stem elongation, BBCH 31–32 to 37–39) may have contributed to lower yield compared with the II season, considering that drought during this period reduces the number of fertile spikelets and grains per spike, while this loss of yield potential is irreversible regardless of environmental conditions in subsequent stages [46].

Elevated temperatures during the II season were associated with accelerated phenological development, a shorter vegetation period, and earlier crop maturation, accompanied by lower variability and earlier attainment of maximum VI values compared with the I season. Similar effects have been reported, showing that higher temperatures can accelerate plant phenological development and maturation, thereby shortening the period during which differences among genotypes can be expressed [5,47,48,49,50]. The shortened vegetation period was accompanied by earlier harvesting and fewer measurements.

The comparison between UAV and POM data acquisition methods clearly indicates their functional differences. The UAV and POM sensors used to calculate the same vegetation indices have different spectral configurations and central wavelengths; therefore, the obtained VI values are not necessarily directly comparable in absolute numerical terms. The comparison between the two sensors was therefore primarily focused on the consistency of their relationships with the investigated agronomic and quality traits, rather than on the assumption that they represent identical spectral measurements. UAV measurements provide integrated high-resolution data and plot-level canopy information that may be advantageous for characterizing yield variability [42,51,52]. In contrast, POM measurements are more sensitive to local physiological differences, which may be relevant for characterizing physiological variation associated with grain quality. These findings are consistent with studies indicating that handheld sensors show stronger relationships for estimating nitrogen content and protein content, whereas UAV measurements better describe the spatial distribution of biomass and yield [53,54].

Furthermore, the results indicate that the relationships between VIs and the investigated traits varied depending on the sensor, phenological stage, genotype group, and growing season. This variability highlights the importance of considering the timing of measurements when interpreting associations between VIs and agronomic and quality traits. Accurate identification of phenological stages is therefore an important prerequisite for interpreting VI–trait relationships, as the strength and direction of these associations may change throughout crop development [44,55].

Comparison across multiple BBCH growth stages and contrasting wheat genotype groups showed that the relationships between VIs and the investigated traits varied with genotype morphology and environmental conditions. These findings further support a context-dependent interpretation of VI-based estimation, indicating that the strength of VI–trait relationships may depend on the combination of genotype group, sensor, measurement stage, and growing season.

Differences between awned and awnless genotypes were also observed, particularly in their relationships with yield. The stronger VI–yield associations observed in awned genotypes may be partly related to the contribution of awns to photosynthetic activity, especially during grain filling when the contribution of leaves may decline [7,8,56]. However, this interpretation should be considered cautiously, as the observed differences may also reflect other genotypic characteristics that were not separately evaluated in this study.

In contrast, awnless genotypes showed relatively stronger associations between VIs and quality traits, particularly protein and wet gluten content. These relationships may reflect differences in photosynthetic activity and nitrogen allocation, which are closely related to grain protein and gluten formation [57,58,59,60]. The stronger associations observed for POM measurements may also indicate the relevance of local physiological variation for grain quality, although this should be interpreted cautiously given the different spatial characteristics of the two sensing approaches. Importantly, the observed differences between awned and awnless groups cannot be attributed exclusively to awn presence, as other genotype-specific characteristics may have contributed to the observed patterns. With nine genotypes per group, the independent effect of awns could not be fully separated. Therefore, these results should be interpreted as differences between genotype groups rather than direct evidence of an independent awn effect [7,8,9]. Further studies with a larger number of genotypes and models explicitly accounting for genotype effects would be useful to clarify these relationships.

The results indicate that NDRE and GNDVI showed potential for assessing variation in grain yield and quality traits, although the strength of these relationships varied depending on the season, genotype group, and measurement conditions. NDRE showed a relatively strong association with grain yield in the corresponding analysis, which is consistent with previous studies highlighting its sensitivity to chlorophyll content and crop density, particularly during later growth stages [60,61]. GNDVI showed associations with both yield and quality parameters, including protein and wet gluten content. During the I season, the comparison of alternative models based on different VIs for protein and wet gluten content showed that GNDVI achieved the best numerical performance; however, its advantage over NDVI and NDRE was not statistically confirmed by direct comparison of model errors (additional model comparison results are provided in the Supplementary Material, File S1). Therefore, the observed performance of GNDVI should be interpreted as an indication of its potential under the conditions examined rather than as evidence of its universal superiority.

The pronounced precipitation deficit during the stem elongation stage (BBCH 31–39) in the I season (17 mm compared with 59 mm in the II season) likely affected canopy structure. Reduced water availability during this stage may cause changes in leaf area and turgor, which can influence the reliability of VIs sensitive to canopy structure and density [62,63]. Under such conditions, GNDVI and NDRE, which are related to chlorophyll content and plant nitrogen status, may reflect crop physiological status differently from NDVI [30]. This may help explain the differences in the strongest VI–yield relationships between seasons: in the I season, GNDVI-A during the heading to the beginning of flowering stage (BBCH 51–61) showed the strongest relationship with yield (r = 0.85), whereas in the II season, the strongest relationship was observed for NDRE-A during the earlier flag leaf stage (BBCH 37–39; r = 0.80). These results suggest that climatic differences between seasons may be associated with differences in yield and in the VI–yield relationships observed at different phenological stages.

Differences between seasons suggest that temperature and precipitation may influence VI dynamics and model performance; however, the individual contributions of these climatic variables were not quantitatively modeled in the present study. The main objective was to evaluate UAV- and POM-derived VIs across phenological stages, genotype groups, and growing seasons rather than to develop an environmental response model. Future studies combining detailed meteorological data with spectral measurements could further clarify the contribution of environmental variability to VI responses and model performance.

The observed relationships with grain yield were generally stronger for UAV-derived VIs, whereas POM-derived VIs showed relevant associations with protein and wet gluten content. These differences may reflect the distinct spatial characteristics of the two sensing approaches, with UAV measurements integrating canopy-level information and POM measurements capturing more localized spectral responses. However, the observed relationships should be interpreted within the experimental conditions and validation framework of the present study.

The linear mixed-effects models indicated that the relationships between VIs and the investigated traits were influenced by both the fixed effects of the vegetation indices and genotype-related variability. For grain yield, the marginal R^2^ values were 0.43 and 0.50 in the I and II seasons, respectively, while the corresponding conditional R^2^ values were 0.78 and 0.64. This indicates that, in addition to the contribution of the VIs, grain yield was associated with a substantial proportion of the explained variation. For protein content, the marginal R^2^ values were 0.30 and 0.39, whereas the conditional R^2^ increased from 0 in the I season to 0.74 in the II season. For wet gluten content, the marginal R^2^ values were 0.33 and 0.31, with conditional R^2^ values of 0.41 and 0.70, respectively. The marked differences between marginal and conditional R^2^, particularly for protein and wet gluten content in the II season, suggest a substantial contribution of genotype-related variability to the observed variation. These findings indicate that VI yield and quality parameter relationships cannot be considered independently of genotype and support a context-dependent interpretation of VI-based estimation rather than a universal relationship applicable across genotypes and seasons.

The leave-one-genotype-out cross-validation (LOGO-CV) provided a more stringent assessment of the ability of the linear mixed-effects models to generalize to genotypes not included in model development. Compared with the model fitting results, the cross-validated RMSE increased for all investigated traits and seasons, with the largest increases observed for grain yield and wet gluten content in the II season. For protein content, the increase in RMSE was relatively small in the I season but more pronounced in the II season. Nevertheless, the LOGO-CV results indicated that the models retained some predictive performance when evaluated on genotypes excluded from model development. These results indicate that model performance was partly dependent on genotype composition and support a context-dependent interpretation of VI-based estimation rather than a universal relationship across wheat genotypes and growing seasons.

The heading to beginning of flowering stage (BBCH 51–61) showed relatively strong relationships between VIs and the investigated yield and quality parameters. At this stage, VI values may still reflect physiological differences among genotypes before the more pronounced chlorophyll degradation associated with later maturation stages, suggesting that measurements at these stages may provide useful information for assessing yield and quality parameters [44,45,51]. In the later stages (grain filling and wax ripeness, BBCH 71–87), weaker VI–yield and VI–quality relationships were generally observed, which may be associated with chlorophyll degradation and crop maturation. However, the most informative VI and measurement stage were not consistent across seasons: GNDVI-A during the heading to beginning of flowering stage (BBCH 51–61) showed the strongest relationship with yield in the I season, whereas NDRE-A during the flag leaf stage (BBCH 37–39) showed the strongest relationship in the II season. This variation may reflect differences in phenological development and environmental conditions between seasons, indicating that VI–yield and VI–quality relationships are dependent on both measurement timing and growing conditions.

The stronger VI–yield relationships observed in awned genotypes may partly be related to the physiological contribution of awns during grain filling, as awns can contribute to spike photosynthesis and carbon assimilation [7,8,9]. However, these differences should be interpreted cautiously, as genotype-specific characteristics may also have contributed to the observed patterns. The stronger relationships observed between POM-derived VIs and grain quality parameters may reflect the sensitivity of proximal measurements to local physiological variation related to nitrogen status and chlorophyll content. Similar potential advantages of proximal optical sensing for assessing crop nutritional status have been reported previously [64].

Overall, the results indicate that the combined use of UAV and POM measurements can provide complementary information for the assessment of wheat yield and grain quality. The relationships between VIs and the investigated parameters varied according to sensing method, phenological stage, genotype group, and growing season, indicating that their estimation performance is context-dependent. Therefore, the contribution of this study lies primarily in the comparative evaluation of these factors under the same experimental conditions rather than in the development of new VIs or prediction algorithms. The LOGO cross-validation further indicated that model performance may depend on genotype composition, supporting cautious interpretation of the obtained relationships. Given the specific agroecological conditions and the limited number of genotypes included in the study, further validation across broader environmental conditions and genetic backgrounds is needed. Nevertheless, the findings provide an exploratory basis for selecting appropriate sensors, VIs, and measurement stages for wheat phenotyping and assessment of grain yield and quality.

Despite the obtained results, several limitations of the present study should be acknowledged. The study was conducted with a relatively limited number of genotypes (18 genotypes), at a single experimental location, and over two growing seasons. Therefore, the findings should be interpreted within the conditions investigated rather than considered broadly generalizable to other genotypes, locations, or environmental conditions. In addition, the absence of an independent external validation dataset limits the extent to which the models can be generalized beyond the experimental population. The repeated observations of the same genotypes were accounted for by applying linear mixed-effects models with genotype as a random effect, while genotype-level LOGO cross-validation was used to assess model generalization, with all observations of a given genotype excluded from model development in each validation step. Differences in temperature, precipitation, and vegetation period between seasons may have affected VI dynamics and their relationships with yield and grain quality. However, the limited number of seasons and single-location design did not allow the effects of genotype, season, and environmental conditions to be fully separated. In addition, the selection of VIs and phenological stages for regression analysis was based on the strength of the observed correlations and was therefore primarily exploratory. This approach may introduce a degree of selection bias, and the results should consequently be regarded as an indication of potential VI–trait relationships under the investigated conditions rather than as evidence of broad predictive applicability. Future studies should also incorporate climatic variables, particularly temperature and precipitation, into the analysis to better quantify their effects on VI dynamics and estimation performance across contrasting growing conditions.

## 4. Materials and Methods

This section presents information on the devices and experimental procedures used during the research.

### 4.1. Experimental Design

**Figure 4:**
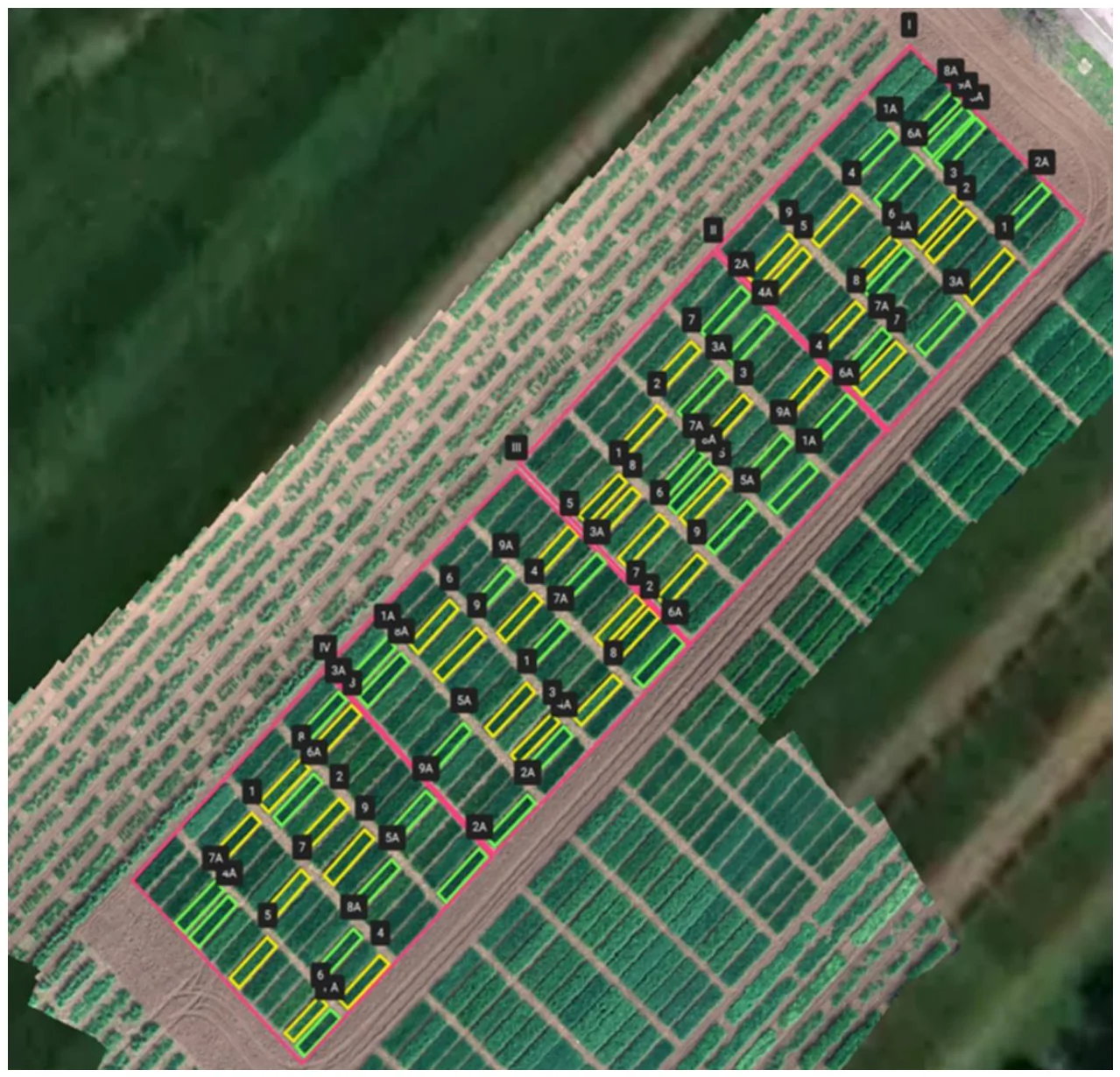
Aerial view of the research experimental design and field layout.

A two-year study was conducted during the 2022/2023 (I season) and 2023/2024 (II season) cropping periods at the experimental field of the Maize Research Institute Zemun Polje, Belgrade (44.86959° N, 20.32903° E). The experiment was established using a randomized block design on chernozem soil type. The research included a total of 72 plots arranged in four blocks (I–IV), with each block comprising 18 plots. Each block contained one plot of each of the 18 investigated genotypes, including nine awned genotypes (1A–9A, Figure 4) and nine awnless genotypes (1–9). Thus, each genotype was represented once in each block, resulting in four replicates per genotype and a total of 72 experimental plots. Individual plots measured 5.2 m^2^. Within each block, the plots were arranged in a randomized order to minimize the potential effects of spatial variability within the experimental area. The 72 plots analyzed in this study were selected from a larger field experiment based on the continuity of cultivation of the same genotypes across both experimental seasons. The same set of genotypes was therefore monitored in both seasons, allowing the vegetation index measurements and agronomic and quality traits to be evaluated under comparable experimental conditions.

The genotypes were classified into awned and awnless groups according to their spike morphology. This grouping was used to distinguish the two genotype groups and to examine whether the relationships between vegetation indices and grain yield and quality traits differed between genotypes with contrasting spike morphologies. Awns are photosynthetically active organs that contribute to spike-level carbon assimilation and may affect canopy light interception and, consequently, multispectral reflectance measurements [7,8,9].

At the studied location, in both research seasons, maize was used as the preceding crop for wheat, and the same production technology was applied. After the maize harvest, residues were shredded, followed by shallow plowing at a depth of 15–20 cm. Before plowing, NPK (6:24:12) mineral fertilizer was applied at a rate of 250 kg∙ha^−1^. Following seedbed preparation, sowing of different wheat genotypes was carried out during the third decade of October at a seeding rate of 500 viable seeds per m^2^, as shown in Figure 4. During the third decade of February, top dressing was performed using UREA mineral fertilizer (46% N) at a rate of 75 kg∙ha^−1^. During the stem elongation stage (beginning of intensive growth, BBCH 31–32), additional fertilizer was applied using ammonium nitrate fertilizer AN (34% N) at a rate of 140 kg∙ha^−1^. Weed and disease control were carried out twice using a combination of Secator 150 mL ha^−1^ (active ingredients: iodosulfuron-methyl-sodium and amidosulfuron) and Acord at 500 mL∙ha^−1^ (active ingredient: glyphosate). Wheat harvest was performed during the second decade of July in the I season and at the beginning of July during the II season. Harvesting was carried out using a WINTERSTEIGER Quantum combine harvester designed for operation on small experimental plots such as those used in this research. Harvest was conducted at full maturity, and the results were standardized and expressed in kg∙ha^−1^.

### 4.2. Technical Equipment

Monitoring of VIs during the research was performed using two devices: a UAV (DJI Phantom 4 Multispectral) and POM (Plant-O-Meter handheld multispectral sensor).

#### 4.2.1. DJI Phantom 4 Multispectral

For aerial imaging, the DJI Phantom 4 Multispectral UAV (DJI, Shenzhen, China [65]) was used, which is capable of detecting reflected solar radiation across multiple spectral bands (visible and near-infrared spectra—NIR). The integrated camera detects six spectral channels: blue (wavelength 450 ± 16 nm), green (wavelength 560 ± 16 nm), red (wavelength 650 ± 16 nm), red edge (wavelength 730 ± 16 nm), near infrared—NIR (wavelength 840 ± 26 nm) and RGB. Flights were conducted under stable weather conditions, without cloud cover or strong wind, during the morning hours (09:00–11:00) in order to ensure uniform illumination conditions. Before each flight, sensor calibration was performed using a reference white calibration panel. The flight altitude was 20 m, enabling high image resolution (~1–3 cm/px). In order to minimize the influence of edge effects, mixed pixels, and potential interference from vegetation in neighboring plots and pathways, a 30% inward buffer was applied. Mean reflectance values and VIs were subsequently calculated from the pixels contained within the resulting polygons. Based on the acquired imagery, VI maps were generated and VI values were calculated using Pix4Dfields (Pix4D) specialized software, version 2.7.0.

#### 4.2.2. Plant-O-Meter

Field VI measurements were additionally performed using a handheld multispectral proximal active sensor called Plant-O-Meter (POM), manufactured by BitGear, Belgrade, Serbia [66]. This optical device is designed with an integrated light source. The device detects light pulses in key spectral regions: blue (455 nm), green (528 nm), red (657 nm), the edge of the red spectrum (740 nm), and two near-infrared—NIR bands (810 nm and 940 nm). This combination enables detailed spectral characterization of vegetation. Thanks to the wide spectral range and high measurement precision, the POM device enables the calculation of a large number of VIs. These calculations are automated using predefined mathematical models, allowing rapid and efficient field analysis. Ambient illumination, such as sunlight, is detected as a direct current component of the signal and removed using a high-pass filter, and only the signal originating from the pulsed operation of the LEDs is retained [67]. In addition, all measurements were conducted at approximately the same time of day and under stable weather conditions in order to further minimize the potential influence of variable illumination on the recorded measurements. Measurements were performed directly above the plant canopy at points uniformly distributed across the observed plots. For each observed plot, 5 to 10 measurements were collected, after which the mean VI value was calculated.

### 4.3. Climatic Conditions of the Observed Years

**Figure 5:**
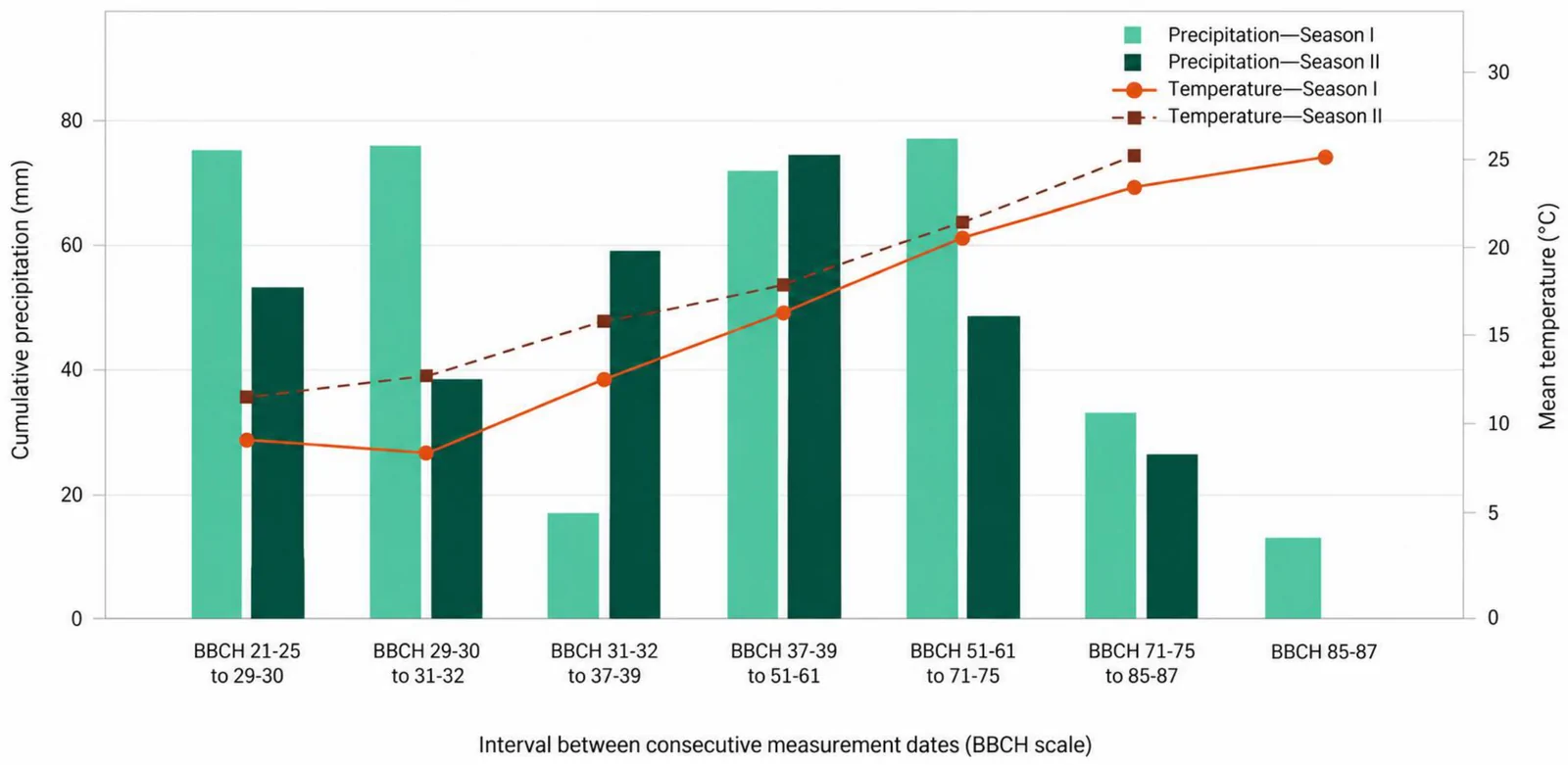
Mean temperature and cumulative precipitation recorded between consecutive measurement dates during Seasons I and II.

Climatic conditions during the I and II seasons showed differences in temperature regime and precipitation amount, which influenced wheat crop development and productivity (Figure 5). The values in Figure 5 represent mean air temperature (°C) and cumulative precipitation (mm) for intervals between consecutive UAV and POM measurement dates corresponding to phenological growth stages according to the BBCH scale. Season II data end at BBCH 71–75 due to earlier harvest. Meteorological data were obtained from the Meteorological Station Zemun Polje, located approximately 200 m from the experimental field.

Season II was characterized by higher temperatures, particularly during the later vegetation stages, which accelerated phenological development and shortened the duration of the growing period. Elevated temperatures during the spring and early summer months of the II season led to earlier crop maturation; therefore, harvest was performed two weeks earlier compared with the previous season. As a result, the number of measurements in the II season was reduced by one measurement. In the I season, a higher precipitation amount was recorded during the early and intermediate crop development stage. The highest precipitation levels were registered in the intervals from BBCH 29–30 to 31–32 and from BBCH 51–61 to 71–75. In contrast, very low precipitation was observed in the I season during the stem elongation–flag leaf stage (BBCH 31–32 to 37–39). Drought during this period reduces the number of fertile spikelets and grains per spike, and this loss of yield potential is irreversible regardless of environmental conditions in the subsequent growth stages [47].

In the II season, the distribution of precipitation was different. The highest amount of precipitation was recorded during the period from BBCH 37–39 to 51–61, corresponding to the middle of the growing season and the period of greatest crop demand.

### 4.4. Stages of Wheat Development

In both observed seasons, measurements were conducted during the following developmental stages: tillering (BBCH 21–25), end of tillering–beginning of stem elongation (BBCH 29–30), stem elongation (BBCH 31–32), flag leaf emergence–end of stem elongation (BBCH 37–39), heading–beginning of flowering (BBCH 51–61), and grain filling (BBCH 71–75). The wax ripeness stage (BBCH 85–87) was omitted in the II season. Crop development dynamics in both seasons showed a high degree of similarity during the early stages (BBCH 21–30), whereas differences became more pronounced during the later development stages (BBCH 51–87).

The tillering and beginning of stem elongation stages (BBCH 21–30) occurred at almost identical times in both seasons, indicating similar environmental conditions during the early vegetation part. However, from the heading–beginning of flowering stage (BBCH 51–61) onward, accelerated crop development was observed in the II season, which was directly associated with higher temperatures during the spring and early summer periods.

### 4.5. Vegetation Indices

Data obtained by remote sensing within the visible (VIS) and near-infrared (NIR) wavelength ranges are used to calculate numerous vegetation indices (VIs), which represent algebraic combinations of reflectance values at different wavelengths [68]. VI values play a key role in monitoring crops, assessing development stages, and identifying problems such as nutrient and water deficiency, as well as detecting pest occurrence. Previous research on the application of VIs in wheat production [42,69,70,71] has shown that VIs can effectively represent wheat crop status and thereby directly contribute to the management of inputs (fertilizer, pesticides, and water) and wheat yield.

**Figure 6:**
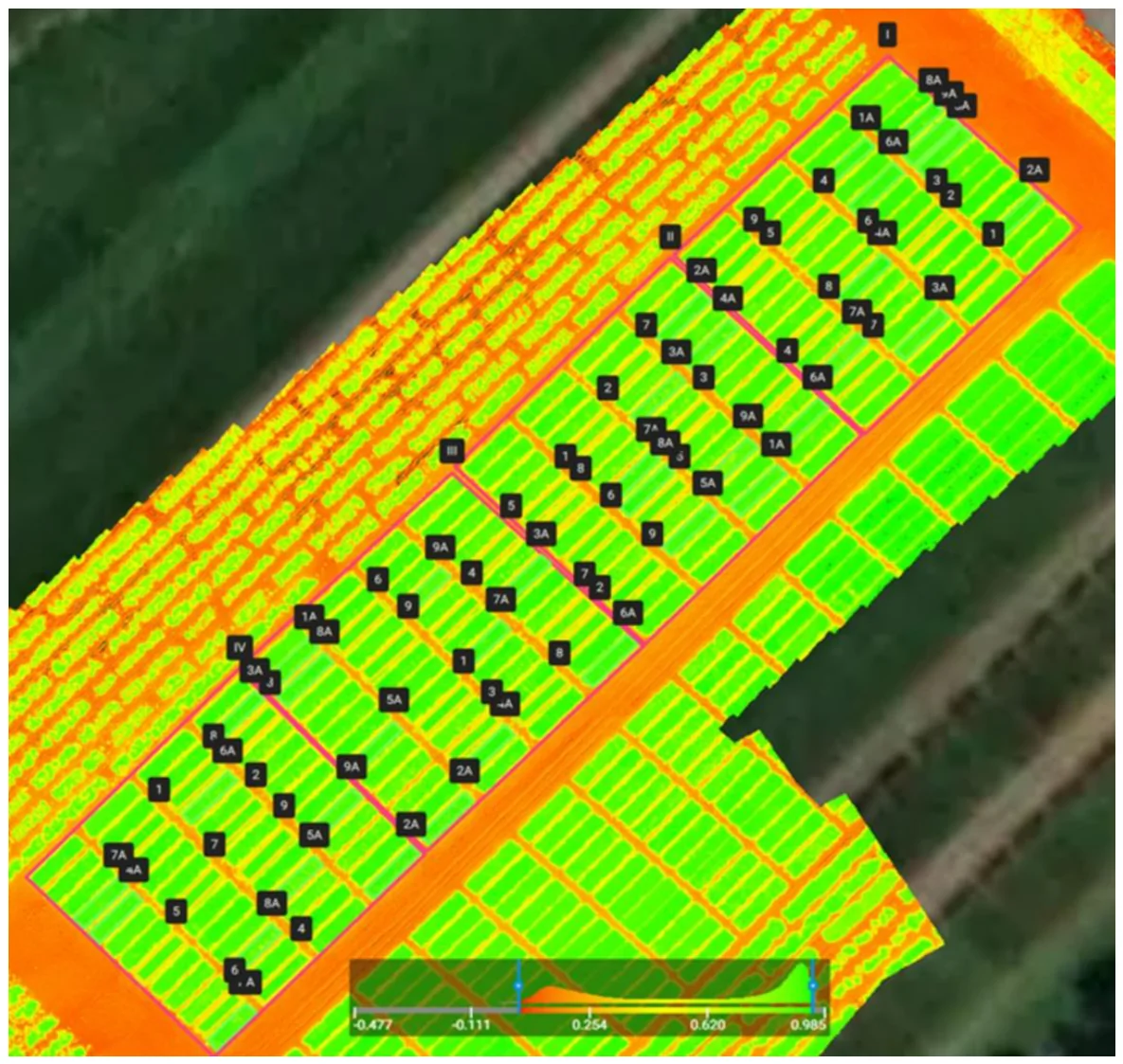
NDVI map of the wheat crop at the experimental site during the I season, first decade of April (BBCH 31–32).

**Figure 7:**
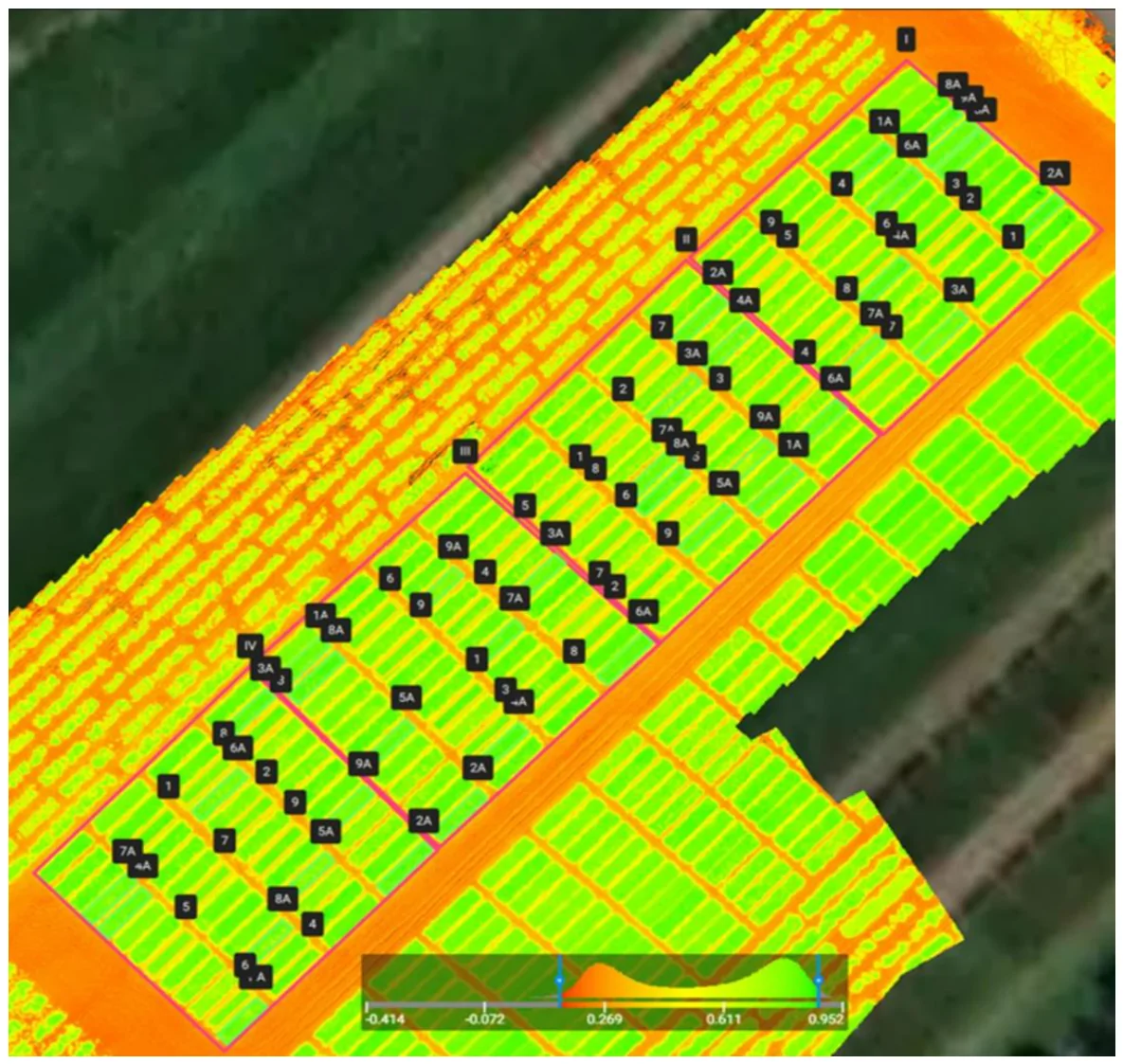
GNDVI map of the wheat crop at the experimental site during the I season, first decade of April (BBCH 31–32).

**Figure 8:**
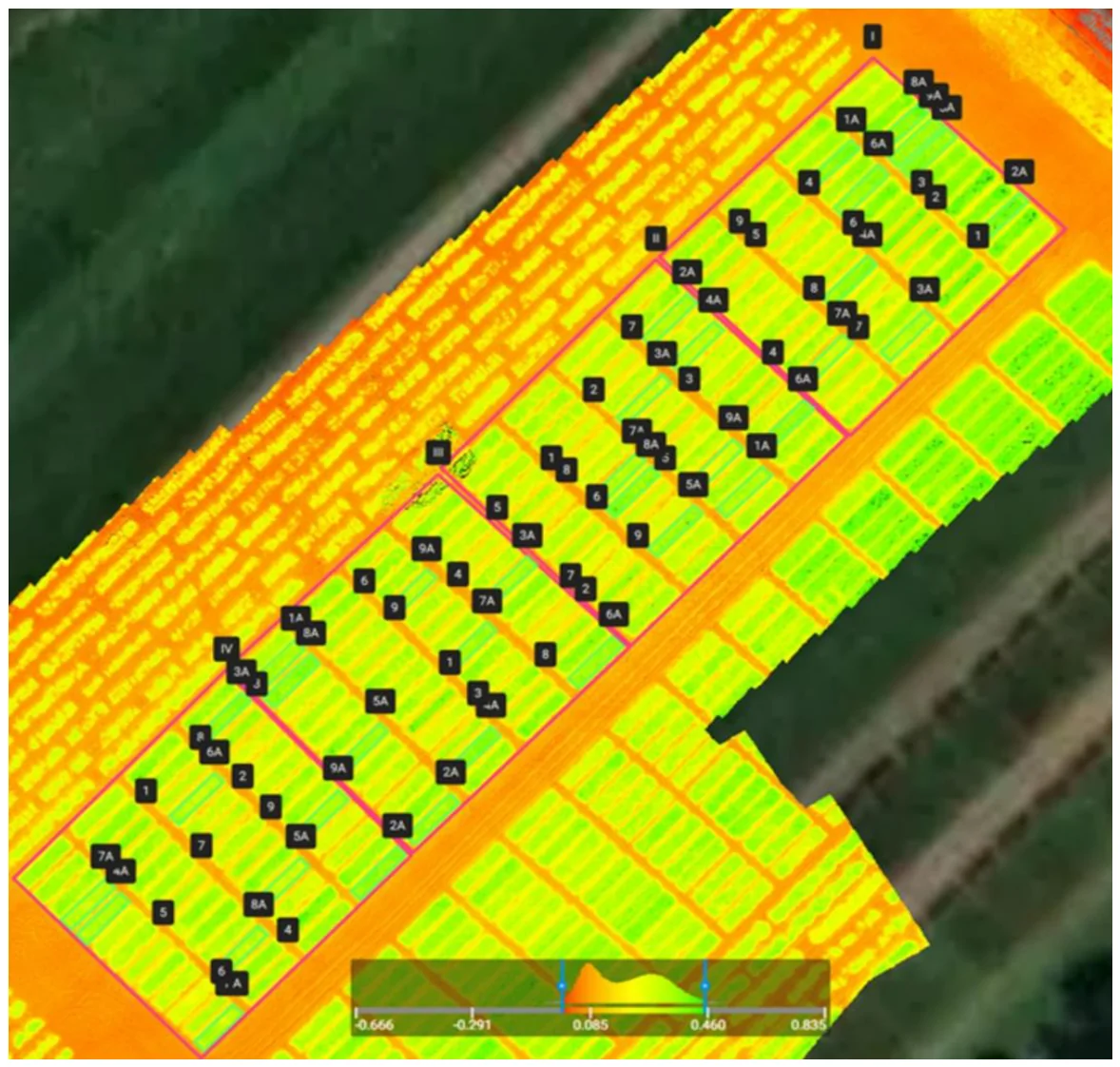
NDRE map of the wheat crop at the experimental site during the I season, first decade of April (BBCH 31–32).

More than twenty VIs were initially derived from the multispectral data. Preliminary correlation analyses were performed between all calculated indices and grain yield, protein content, and wet gluten content. Based on these analyses, NDVI, GNDVI, and NDRE (Figure 6, Figure 7 and Figure 8) were selected for detailed investigation, as they consistently showed the strongest relationships with the investigated traits across different phenological stages and both growing seasons. Furthermore, these indices represent complementary spectral responses associated with canopy biomass, chlorophyll and nitrogen status, and red-edge reflectance, respectively.

#### 4.5.1. NDVI

NDVI is calculated based on the reflectance of red and near-infrared light [72,73,74]. According to Equation (1) [75], NDVI ranges from −1 to +1, where values close to +1 indicate healthy vegetation, values between 0 and 0.2 generally correspond to bare soil or dry vegetation, and negative values indicate the presence of water or snow [76].

(1) $$NDVI=\frac{\left(NIR-RED\right)}{\left(NIR+RED\right)}$$

#### 4.5.2. GNDVI

GNDVI represents a modified form of NDVI in which the red band is replaced by the green band [77]. GNDVI values are calculated using Equation (2) [78] and range from −1 to +1, where values between 0.1 and 0.5 indicate poorly developed crops or crops affected by certain stress factors. Values above 0.5 are generally associated with healthy and well-developed vegetation [79].

(2) $$GNDVI=\frac{\left(NIR-GREEN\right)}{\left(NIR+GREEN\right)}$$

#### 4.5.3. NDRE

NDRE generally shows lower absolute values compared with NDVI and GNDVI. NDRE values are calculated using Equation (3) [80] and range from −1 to 0.6. For wheat crops, NDRE values commonly range between 0.1 and 0.4, while values close to 0.6 are rarely reached. Values between 0 and 0.2 indicate weak vegetation, whereas values from 0.2 to 0.4 indicate moderately developed crops. Values above 0.4 are associated with dense and healthy vegetation [81].

(3) $$NDRE=\frac{\left(NIR-RedEdge\right)}{\left(NIR+RedEdge\right)}$$

### 4.6. Wheat Yield, Protein Content, and Wet Gluten Content

In addition to tracking VIs during vegetation, the research also analyzed wheat yield and its main quality indicators, namely, protein and wet gluten content.

Final grain yield was measured directly in the field by determining the total grain mass per observed plot after harvest. Harvest was performed at full maturity (BBCH 85–87). The obtained results were standardized and expressed per hectare (kg∙h^−1^). To determine protein and wet gluten content, 500 g wheat samples were collected from each plot. The samples were subsequently analyzed using the Infraneo N.I.R./T. analyzer (CHOPIN TECHNOLOGIES, France), a specialized instrument for measuring protein and wet gluten content in wheat. This method enables precise and rapid determination of both parameters, providing valuable information on wheat grain quality. Wet gluten content includes water retained within the gluten matrix in addition to gluten proteins, which explains why wet gluten values are typically higher than total grain protein content (Table 1). These analyses contribute to the assessment of the nutritional value of wheat and its suitability for different uses, and they provide key information for crop management, selection, and development of new genotypes.

### 4.7. Statistical Analysis

Descriptive statistics, including mean values, standard deviations, and coefficient of variation, were calculated for all traits and device-derived VIs across all phenological stages and both growing seasons.

Prior to correlation and regression analyses, the normality of the data distribution was assessed using the Shapiro–Wilk test. Since no significant deviations from normality were detected (*p* > 0.05), parametric statistical methods, including Pearson’s correlation coefficient and linear regression analyses, were applied.

The interdependence between VIs and yield parameters was analyzed using Pearson’s correlation coefficient (r). Correlation analyses were conducted separately for awned and awnless wheat genotypes in order to determine differences in their responses. Correlations were calculated between NDVI, GNDVI, and NDRE obtained from the UAV and POM devices and yield, protein content, and wet gluten content. Correlation analyses were performed for each observed development stage, enabling assessment of the dynamics of the relationship between VIs and the analyzed grain yield and quality parameters.

For the most pronounced statistically significant correlations, linear mixed-effects regression analysis was additionally applied. For each observed variable (yield, protein content, and wet gluten content), the vegetation index showing the strongest correlation was selected for further analysis, together with the corresponding sensor and phenological stage. In the linear mixed-effects models, the selected VI was included as a fixed effect, while genotype was included as a random effect to account for the hierarchical structure of the data and the repeated measurements within genotypes. Statistical significance was evaluated at the *p* < 0.05 and *p* < 0.01 levels.

The performance of the linear mixed-effects models was evaluated using the marginal coefficient of determination (R^2^m), representing the proportion of variance explained by the fixed effect, and the conditional coefficient of determination (R^2^c), representing the proportion of variance explained by the entire model, including both fixed and random effects. The estimated regression coefficient (β) and its 95% confidence interval (95% CI) were also calculated. Model significance was assessed using the F-statistic and corresponding *p*-value. Additional performance metrics, including root mean square error (RMSE), mean absolute error (MAE), relative error, bias, Nash–Sutcliffe efficiency (NSE), and Lin’s concordance correlation coefficient (CCC), were calculated to provide a comprehensive assessment of model estimation performance.

To assess the robustness and generalization ability of the obtained linear mixed-effects models, a leave-one-genotype-out (LOGO) cross-validation procedure was applied. In each iteration, all observations belonging to one genotype were excluded, and the model was fitted using the remaining genotypes. The fitted model was subsequently used to estimate the values for the excluded genotype. This procedure was repeated sequentially until each genotype had been excluded once and used as an independent validation set. Based on the observed and estimated values obtained across all validation iterations, the coefficient of determination (R^2^LOGO), root mean square error of cross-validation (RMSELOGO), and mean absolute error of cross-validation (MAELOGO) were calculated.

Statistical analyses were conducted using IBM SPSS Statistics 25 and R programming language (version 4.6.0). Linear mixed-effects models were fitted using the lme4 package, while correlation heatmaps were generated using the ggplot2 package.

## 5. Conclusions

The results of this study indicate that UAV- and POM-derived vegetation indices can provide useful information for the assessment of wheat grain yield and quality, although their relationships with the investigated parameters varied according to sensing method, vegetation index, phenological stage, genotype group, and growing season.

The observed differences among the awned and awnless genotype groups suggest that genotype characteristics should be considered when interpreting VI–yield and VI–quality relationships. The linear mixed-effects models further showed that genotype-related variability contributed substantially to the observed variation in several investigated parameters, highlighting the importance of accounting for genotype effects in VI-based estimation.

The VI showing the strongest observed yield relationship differed between seasons, with GNDVI-A during BBCH 51–61 in the I season and NDRE-A during BBCH 37–39 in the II season, indicating that the most informative index and measurement stage may vary under different growing conditions. Relationships with protein and wet gluten content were generally more variable, particularly when evaluated using genotype-level LOGO cross-validation.

Overall, the findings support the use of multispectral sensing as a potential tool for exploratory assessment and phenotyping of wheat yield and grain quality while emphasizing that VI-based relationships are context-dependent. Within the conditions investigated, UAV- and POM-derived measurements may provide complementary information for research and breeding applications. However, the results should not be interpreted as evidence of universal predictive applicability. The study was conducted using 18 genotypes, two growing seasons, and a single experimental location; therefore, further research involving a larger and more diverse set of genotypes, multiple locations, and additional growing seasons is needed to evaluate the robustness and transferability of the observed relationships.

## Figures and Tables

**Table 1:** Descriptive statistics of the observed parameters (grain yield, protein content, and wet gluten content).

Parameters	Statistics	I Season		II Season	
		Awned	Awnless	Awned	Awnless
Yield (kg∙ha^−1^)	Mean	5337.82	5215.01	11,728.75	11,505.04
	SD	1013.10	745.51	1391.85	1099.68
	Cv	18.98	14.29	11.87	9.55
Protein Content (%)	Mean	10.85	10.96	11.69	11.41
	SD	0.93	0.79	0.84	1.07
	Cv	8.53	7.18	7.16	9.37
Wet Gluten Content (%)	Mean	19.11	20.00	24.14	23.55
	SD	2.99	2.64	2.41	3.47
	Cv	15.64	13.20	9.98	14.73

Notes: Mean—mean value, SD—standard deviation, and Cv—coefficient of variation (%).

**Table 2:** Descriptive statistics of VIs obtained using UAV measurements during the I season.

Wheat Phenological Development Stages	Statistics	NDVI—A	NDVI	GNDVI—A	GNDVI	NDRE—A	NDRE
(BBCH 21–25)	Mean	0.66	0.64	0.56	0.55	0.15	0.14
	SD	0.05	0.06	0.06	0.04	0.01	0.01
	Cv	7.60	9.32	10.66	7.24	6.89	6.90
(BBCH 29–30)	Mean	0.79	0.79	0.68	0.67	0.21	0.21
	SD	0.03	0.04	0.03	0.03	0.02	0.02
	Cv	3.79	5.09	4.46	4.46	9.34	9.43
(BBCH 31–32)	Mean	0.86	0.84	0.72	0.71	0.25	0.24
	SD	0.03	0.03	0.03	0.03	0.03	0.03
	Cv	3.50	3.55	4.15	4.21	11.85	11.88
(BBCH 37–39)	Mean	0.86	0.86	0.75	0.74	0.28	0.28
	SD	0.03	0.03	0.04	0.03	0.03	0.03
	Cv	3.48	3.48	5.34	4.06	10.60	10.71
(BBCH 51–61)	Mean	0.68	0.67	0.63	0.62	0.22	0.22
	SD	0.06	0.05	0.05	0.04	0.03	0.02
	Cv	8.81	7.48	7.94	6.51	13.10	9.01
(BBCH 71–75)	Mean	0.34	0.33	0.41	0.41	0.12	0.12
	SD	0.05	0.03	0.03	0.02	0.01	0.01
	Cv	14.66	9.17	7.28	4.84	8.33	8.4
(BBCH 85–87)	Mean	0.21	0.22	0.32	0.34	0.10	0.11
	SD	0.05	0.04	0.03	0.03	0.01	0.01
	Cv	23.25	18.26	9.23	8.84	10.10	9.09

Notes: Mean—mean value, SD—standard deviation, and Cv—coefficient of variation (%).

**Table 3:** Descriptive statistics of VIs obtained using POM during the I season

Wheat Phenological Development Stages	Statistics	NDVI—A	NDVI	GNDVI—A	GNDVI	NDRE—A	NDRE
(BBCH 21–25)	Mean	0.60	0.58	0.59	0.58	0.11	0.11
	SD	0.06	0.06	0.03	0.03	0.01	0.01
	Cv	9.97	10.42	5.06	5.22	8.55	8.85
(BBCH 29–30)	Mean	0.63	0.64	0.63	0.64	0.16	0.17
	SD	0.05	0.04	0.04	0.03	0.02	0.02
	Cv	7.93	6.25	6.39	4.81	12.34	11.98
(BBCH 31–32)	Mean	0.77	0.79	0.69	0.70	0.21	0.20
	SD	0.04	0.04	0.03	0.03	0.02	0.02
	Cv	5.18	5.08	4.33	4.28	9.61	9.95
(BBCH 37–39)	Mean	0.70	0.72	0.64	0.65	0.18	0.17
	SD	0.05	0.05	0.05	0.05	0.04	0.04
	Cv	7.10	6.93	7.81	7.69	22.22	23.52
(BBCH 51–61)	Mean	0.522	0.514	0.519	0.518	0.124	0.121
	SD	0.06	0.06	0.04	0.04	0.03	0.03
	Cv	11.49	11.67	7.7	7.72	24.19	24.79
(BBCH 71–75)	Mean	0.22	0.21	0.35	0.35	0.07	0.07
	SD	0.04	0.04	0.03	0.03	0.02	0.02
	Cv	17.7	18.69	8.52	8.62	27.78	29.85
(BBCH 85–87)	Mean	0.19	0.18	0.33	0.33	0.06	0.05
	SD	0.07	0.07	0.05	0.04	0.02	0.02
	Cv	36.46	38.89	15.10	12.08	33.33	39.69

Notes: Mean—mean value, SD—standard deviation, and Cv—coefficient of variation (%).

**Table 4:** Descriptive statistics of VIs obtained using UAV measurements during the II season.

Wheat Phenological Development Stages	Statistics	NDVI—A	NDVI	GNDVI—A	GNDVI	NDRE—A	NDRE
(BBCH 21–25)	Mean	0.81	0.81	0.67	0.69	0.24	0.24
	SD	0.03	0.03	0.03	0.03	0.02	0.02
	Cv	3.7	3.7	4.36	4.33	8.51	8.3
(BBCH 29–30)	Mean	0.89	0.89	0.79	0.79	0.33	0.32
	SD	0.02	0.02	0.02	0.02	0.03	0.03
	Cv	2.24	2.24	2.52	2.53	8.45	8.7
(BBCH 31–32)	Mean	0.91	0.90	0.82	0.81	0.39	0.38
	SD	0.01	0.01	0.02	0.02	0.03	0.03
	Cv	1.10	1.11	2.40	2.47	7.61	7.94
(BBCH 37–39)	Mean	0.9	0.89	0.82	0.81	0.41	0.38
	SD	0.02	0.01	0.02	0.02	0.03	0.03
	Cv	2.22	1.13	2.43	2.47	7.31	7.81
(BBCH 51–61)	Mean	0.82	0.81	0.73	0.73	0.32	0.31
	SD	0.04	0.03	0.04	0.04	0.04	0.04
	Cv	4.89	3.72	5.48	5.51	12.54	12.78
(BBCH 71–75)	Mean	0.27	0.28	0.45	0.46	0.13	0.13
	SD	0.03	0.03	0.03	0.03	0.01	0.01
	Cv	11.15	10.79	6.68	6.56	10.77	9.77

Notes: Mean—mean value, SD—standard deviation, and Cv—coefficient of variation (%).

**Table 5:** Descriptive statistics of VIs obtained using POM during the II season.

Wheat Phenological Development Stages	Statistics	NDVI—A	NDVI	GNDVI-A	GNDVI	NDRE—A	NDRE
(BBCH 21–25)	Mean	0.70	0.69	0.67	0.66	0.19	0.19
	SD	0.04	0.05	0.03	0.03	0.02	0.02
	Cv	5.75	7.29	4.48	4.51	8.77	8.23
(BBCH 29–30)	Mean	0.79	0.80	0.72	0.73	0.23	0.32
	SD	0.04	0.04	0.03	0.03	0.03	0.03
	Cv	5.09	4.97	4.16	4.10	13.27	9.43
(BBCH 31–32)	Mean	0.76	0.77	0.71	0.71	0.25	0.24
	SD	0.04	0.03	0.03	0.03	0.03	0.04
	Cv	5.28	3.92	4.23	4.20	11.65	15.38
(BBCH 37–39)	Mean	0.73	0.72	0.68	0.68	0.24	0.22
	SD	0.03	0.04	0.02	0.03	0.03	0.02
	Cv	4.13	4.91	3.36	4.94	11.86	8.58
(BBCH 51–61)	Mean	0.66	0.63	0.64	0.62	0.19	0.19
	SD	0.05	0.04	0.03	0.03	0.03	0.03
	Cv	7.62	6.34	4.71	4.88	15.38	15.46
(BBCH 71–75)	Mean	0.14	0.14	0.37	0.36	0.08	0.08
	SD	0.03	0.02	0.03	0.03	0.01	0.01
	Cv	21.12	14.71	8.06	8.40	12.50	12.35

Notes: Mean—mean value, SD—standard deviation, and Cv—coefficient of variation (%).

**Table 6:** Summary of linear mixed-effects regression models.

Variables	Season	BBCH	Sensor	VIs	N	R^2^m	R^2^c	β (95% CI)	F	*p*
Yield	I	51–61	UAV	GNDVI-A	36	0.43	0.78	10,453.92 (5456.00–15,451.60)	16.81	7.20 × 10^−4^
	II	37–39	UAV	NDRE-A	36	0.50	0.64	30,464.56 (19,269.20–41,659.92	28.44	4.53 × 10^−5^
PC	I	51–61	POM	GNDVI	36	0.30	0.00	8.96 (4.43–13.48)	15.06	4.56 × 10^−4^
	II	51–61	POM	GNDVI	36	0.39	0.74	19.99 (13.02–26.98)	31.54	3.35 × 10^−6^
WGC	I	51–61	POM	GNDVI	36	0.33	0.41	31.30 (15.79–46.8)	15.65	4.56 × 10^−4^
	II	51–61	POM	GNDVI	36	0.31	0.70	57.92 (33.83–82.09)	22.21	4.58 × 10^−5^

Notes: PC—protein content, WGC—wet gluten content, BBCH—phenological development stages, UAV—Unmanned Aerial Vehicle, POM—Plant-O-Meter, N—sample size, R^2^m—marginal coefficient of determination, R^2^c—conditional coefficient of determination, β—fixed-effect regression coefficient (kg∙ha^−1^/%), CI—confidence interval (kg∙ha^−1^/%), F value—F statistic model, and *p*-value—significance level.

**Table 7:** Performance metrics of the linear mixed-effects models.

Variables	Season	BBCH	VIs	N	RMSE	MAE	RE	Bias	NSE	Lin’s CCC
Yield	I	51–61	GNDVI-A	36	349.79	276.68	5.72	0	0.88	0.93
	II	37–39	NDRE-A	36	674.08	493.12	4.54	0	0.76	0.86
PC	I	51–61	GNDVI	36	0.65	0.53	4.94	0	0.31	0.47
	II	51–61	GNDVI	36	0.46	0.39	3.43	0	0.81	0.89
WGC	I	51–61	GNDVI	36	1.92	1.52	7.85	0	0.45	0.60
	II	51–61	GNDVI	36	1.59	1.36	5.83	0	0.78	0.87

Notes: PC—protein content, WGC—wet gluten content, BBCH—phenological development stages, N—sample size, RMSE—root mean square error (kg∙ha^−1^/%), MAE—mean absolute error (kg∙ha^−1^/%), RE—relative error (%), Bias—mean prediction bias, NSE—Nash–Sutcliffe efficiency, and Lin’s CCC—Lin’s concordance correlation coefficient.

**Table 8:** Leave-one-genotype-out cross-validation performance of the linear mixed-effects models.

Variables	Years	VIs	R^2^LOGO	RMSELOGO	MAELOGO
Yield	I	GNDVI-A	0.53	732.09	597.28
	II	NDRE-A	0.51	971.58	720.57
PC	I	GNDVI	0.26	0.67	0.58
	II	GNDVI	0.38	0.84	0.69
WGC	I	GNDVI	0.25	2.27	2.00
	II	GNDVI	0.31	2.86	2.45

Notes: PC—protein content, WGC—wet gluten content, R^2^LOGO—coefficient of determination obtained by leave-one-genotype-out cross-validation, RMSELOGO—root mean square error of leave-one-genotype-out cross-validation (kg∙ha^−1^/%), and MAELOGO—mean absolute error of leave-one-genotype-out cross-validation (kg∙ha^−1^/%).

## Data Availability

Data are contained within the article.

## Supplementary Materials

The following supporting information can be downloaded at: https://www.mdpi.com/article/10.3390/plants15182819/s1, Figure S1: Regression models for grain yield estimation with 95% prediction intervals: (A) GNDVI-A in Season I and (B) NDRE-A in Season II. Figure S2: Regression models for protein content estimation with 95% prediction intervals: (A) GNDVI in Season I and (B) GNDVI in Season II. Figure S3: Regression models for gluten content estimation with 95% prediction intervals: (A) GNDVI in Season I and (B) GNDVI in Season II. Figure S4: Observed-versus-estimated values and residual distributions for six models. (A) GNDVI-A—yield, Season I (B) NDRE-A—yield, Season II (C) GNDVI—protein content, Season I (D) GNDVI—protein content, Season II, (E) GNDVI—wet gluten content, Season I (F) GNDVI—wet gluten content, Season II. Supplementary File S1: Comparison of Regression Models Based on NDVI, GNDVI, and NDRE.

## Abbreviations

The following abbreviations are used in this manuscript:

VIs	Vegetation indices
NDVI	Normalized Difference Vegetation Index
GNDVI	Green Normalized Difference Vegetation Index
NDRE	Normalized Difference Red Edge Index
BBCH	Biologische Bundesanstalt Bundessortenamt and CHemical industry
UAV	Unmanned Aerial Vehicle
POM	Plant-O-Meter handheld sensor
PC	Protein content
WGC	Wet gluten content
NIR	Near infrared spectrum regions
VIS	Visible spectrum regions
LOGO	Leave-one-genotype-out
