# Supplementary material for: Estimation of Grain Yield and Quality in Awned and Awnless Wheat Genotypes Using UAV and Proximal Multispectral Sensors
Source: Plants (Basel). 2026 Sep 14;15(18):2819. doi: 10.3390/plants15182819 (PMC13610374; doi:10.3390/plants15182819)
Supplement: Supplementary file 1 [file plants-15-02819-s001.zip › File S1. Comparison of Regression Models Based on NDVI, GNDVI, and NDRE.pdf]

Comparison of Regression Models Based on NDVI, GNDVI, and NDRE

To further assess whether differences in model performance based on different vegetation indices represent an actual advantage of one index over the others, direct comparisons of the regression models were performed. In addition to the coefficient of determination (R^2), residual standard error, and information criteria (AIC and BIC), the absolute prediction errors were compared using the Wilcoxon signed-rank test for paired samples. This approach allows a distinction to be made between the **numerically best-performing model** and a model whose advantage is statistically supported.

First growing season – grain protein content

For the estimation of grain protein content in the first growing season, linear regression models based on NDVI, GNDVI, and NDRE were compared. All three vegetation indices showed a statistically significant association with protein content. GNDVI achieved the highest ability to explain the variability in protein content (R^2 = 0.307; β = 8.956; p = 0.000456), compared with NDVI (R^2 = 0.212; β = 5.844; p = 0.00474) and NDRE (R^2 = 0.115; β = 8.478; p = 0.0434). The GNDVI model also had the lowest residual standard error (0.665), compared with NDVI (0.709) and NDRE (0.752).

Comparison based on the information criteria further supported the GNDVI model, which had the lowest AIC (76.75) and BIC (81.50) values, whereas the corresponding values for NDVI were 81.38 and 86.13, and for NDRE 85.56 and 90.31. However, direct comparison of the absolute prediction errors using the Wilcoxon signed-rank test for paired samples revealed no statistically significant differences between the GNDVI and NDVI models (p = 0.3542), or between the GNDVI and NDRE models (p = 0.3877). Therefore, although GNDVI showed the best numerical performance, characterized by the highest R^2, lowest residual error, and lowest AIC and BIC values, its advantage over NDVI and NDRE was not statistically confirmed based on the direct comparison of prediction errors.

First growing season – grain gluten content

For the estimation of grain gluten content in the first growing season, linear regression models based on NDVI, GNDVI, and NDRE were compared. GNDVI showed the highest ability to explain the variability in gluten content (R^2 = 0.337; β = 31.460; p = 0.000206), compared with NDVI (R^2 = 0.259; β = 21.667; p = 0.001525) and NDRE (R^2 = 0.082; β = 24.023; p = 0.0906). The GNDVI model also had the lowest residual standard error (2.181), whereas the corresponding values for NDVI and NDRE were 2.306 and 2.567, respectively.

Comparison based on the information criteria further supported the GNDVI model, which had the lowest AIC (162.26) and BIC (167.01) values, compared with NDVI (166.26 and 171.01) and NDRE (173.98 and 178.73). However, direct comparison of the absolute prediction errors did not reveal statistically significant differences between the GNDVI and NDVI models (p = 0.3461), or between the GNDVI and NDRE models (p = 0.3792). Therefore, although GNDVI showed the best numerical performance for estimating grain gluten content in the first growing season, its advantage over NDVI and NDRE was not statistically confirmed based on the direct comparison of prediction errors.

Second growing season – awnless genotypes, grain protein content

For the estimation of grain protein content in the second growing season for awnless genotypes, linear regression models based on NDVI, GNDVI, and NDRE were compared. All three vegetation indices showed a statistically significant association with protein content. GNDVI showed the strongest effect ($\beta = 19.999$; SE = 3.561; $p = 3.35 \times 10^{-6}$), while NDVI and NDRE were also statistically significant ($\beta = 15.742$; SE = 3.128; $p = 1.68 \times 10^{-5}$ and $\beta = 18.487$; SE = 6.325; $p = 0.00614$, respectively).

The GNDVI model showed the lowest residual standard deviation (0.528), compared with NDVI (0.557) and NDRE (0.661). Comparison based on the information criteria further supported the GNDVI model, which had the lowest AIC (78.60) and BIC (84.93) values, whereas the corresponding values for NDVI were 82.16 and 88.50, and for NDRE 94.11 and 100.45. Direct comparison of the absolute prediction errors using the Wilcoxon signed-rank test for paired samples showed that the difference between the GNDVI and NDVI models was not statistically significant ($p = 0.1568$), whereas the GNDVI model had significantly lower prediction errors than the NDRE model ($p = 0.01528$). Thus, GNDVI showed the best overall numerical performance, with a statistically supported advantage over NDRE, whereas its difference from NDVI was not statistically significant.

Second growing season – awnless genotypes, grain gluten content

For the estimation of grain gluten content in the second growing season for awnless genotypes, models based on NDVI, GNDVI, and NDRE were compared. GNDVI showed the highest ability to explain the variability in gluten content ($\beta = 57.919$; SE = 12.291; $p = 4.58 \times 10^{-5}$), compared with NDVI ($\beta = 43.546$; SE = 10.864; $p = 0.00033$) and NDRE ($\beta = 51.321$; SE = 20.786; $p = 0.01873$). The GNDVI model had the lowest residual standard deviation (SD = 1.824), whereas the corresponding values for NDVI and NDRE were 1.933 and 2.135, respectively.

Comparison based on the information criteria further supported the GNDVI model, which had the lowest AIC (167.66) and BIC (174.00) values, compared with NDVI (AIC = 171.82; BIC = 178.16) and NDRE (AIC = 179.85; BIC = 186.19). Direct comparison of the absolute prediction errors showed that the GNDVI model had significantly lower errors than the NDVI model (Wilcoxon signed-rank test for paired samples, $p = 0.04088$), whereas the difference between the GNDVI and NDRE models was not statistically significant ($p = 0.1348$). Based on these results, GNDVI showed the best overall performance for estimating grain gluten content, with a statistically supported advantage over NDVI, whereas its advantage over NDRE did not reach statistical significance.
